# Metatranscriptome Library Preparation Influences Analyses of Viral Community Activity During a Brown Tide Bloom

**DOI:** 10.3389/fmicb.2021.664189

**Published:** 2021-05-31

**Authors:** Eric R. Gann, Yoonja Kang, Sonya T. Dyhrman, Christopher J. Gobler, Steven W. Wilhelm

**Affiliations:** ^1^Department of Microbiology, University of Tennessee, Knoxville, Knoxville, TN, United States; ^2^Department of Ocean Integrated Science, School of Marine Technology, Chonnam National University, Yeosu, South Korea; ^3^Biology and Paleo Environment Division, Lamont-Doherty Earth Observatory, Columbia University, New York, NY, United States; ^4^Department of Earth and Environmental Sciences, Columbia University, New York, NY, United States; ^5^School of Marine and Atmospheric Sciences, Stony Brook University, Stony Brook, NY, United States

**Keywords:** library preparation, rRNA reduction, polyadenylation selection, virus ecology, marine microbiology

## Abstract

There is growing interest in the use of metatranscriptomics to study virus community dynamics. We used RNA samples collected from harmful brown tides caused by the eukaryotic alga *Aureococcus anophagefferens* within New York (United States) estuaries and in the process observed how preprocessing of libraries by either selection for polyadenylation or reduction in ribosomal RNA (rRNA) influenced virus community analyses. As expected, more reads mapped to the *A. anophagefferens* genome in polyadenylation-selected libraries compared to the rRNA-reduced libraries, with reads mapped in each sample correlating to one another regardless of preprocessing of libraries. Yet, this trend was not seen for reads mapping to the Aureococcus anophagefferens Virus (AaV), where significantly more reads (approximately two orders of magnitude) were mapped to the AaV genome in the rRNA-reduced libraries. In the rRNA-reduced libraries, there was a strong and significant correlation between reads mappings to AaV and *A. anophagefferens*. Overall, polyadenylation-selected libraries produced fewer viral contigs, fewer reads mapped to viral contigs, and different proportions across viral realms and families, compared to their rRNA-reduced pairs. This study provides evidence that libraries generated by rRNA reduction and not selected for polyadenylation are more appropriate for quantitative characterization of viral communities in aquatic ecosystems by metatranscriptomics.

## Introduction

Viruses are important modulators of aquatic microbial communities. This includes structuring communities through lysis of dominant members (Thingstad, 2000; Pound et al., 2020), shunting organic nutrients for uptake by the microbial community (Wilhelm and Suttle, 1999), or through significantly altering infected cells metabolism (Rosenwasser et al., 2016; Zimmerman et al., 2020), causing the production of novel virus–encoded compounds. For example, infections can increase the release of complex polysaccharides (Nissimov et al., 2018) that may be used by heterotrophic microbial communities (Alderkamp et al., 2007; Bar-Zeev and Rahav, 2015). Changes in metabolism can also alter sinking and aggregate formation (Lawrence and Suttle, 2004; Yamada et al., 2018), modulating availability of nutrients by shuttling carbon into the deep ocean, in contrast to the viral shunt (Sullivan et al., 2017; Laber et al., 2018). Since the discovery that viruses are the most abundant biological entities in the ocean (Bergh et al., 1989; Proctor and Fuhrman, 1990; Suttle et al., 1990), these roles and the dynamics of viruses in aquatic microbial communities have been investigated extensively. Microscopy has been used to understand changes in total virus particle communities by both epifluorescence staining (Proctor and Fuhrman, 1990) and electron microscopy (Gastrich et al., 2004). Once information regarding a subset of the virus community is known, both culture-dependent and culture-independent approaches have been used. Challenging known hosts with water samples and performing most probable number assays (Tarutani et al., 2000), or plaque assays (Bellec et al., 2010), have assessed infectious particles, whereas polymerase chain reaction (PCR) amplicon sequencing (Moniruzzaman et al., 2016) and quantitative PCR–based (Short et al., 2011) approaches have assessed specific members without the need of culturing. More recently, metagenomic (Wilson et al., 2017) and metatranscriptomic (Moniruzzaman et al., 2017) approaches have evolved to provide information on the dynamics, composition, and activity of viral communities in aquatic systems.

Viruses of phytoplankton are hypothesized to contribute to algal bloom collapse in many systems (Tarutani et al., 2000; Gastrich et al., 2004; Vardi et al., 2012). The pelagophyte *Aureococcus anophagefferens* causes brown tide blooms in shallow bays, globally, causing millions of dollars in losses, due to the severe light attenuation caused by high cell densities and blooms potentially producing compounds toxic to bivalves (Gobler and Sunda, 2012). These blooms occur as *A. anophagefferens* can outcompete other photosynthetic members in the water column when inorganic nutrients are low (Gobler and Sunda, 2012; Yao et al., 2019). It has long been hypothesized that viruses play an important role in brown tide bloom termination, as natural populations of *A. anophagefferens* were visualized to be infected with viruses since the first characterization of these blooms (Sieburth et al., 1988). Moreover, during bloom collapse, the percentage of cell infected with viruses has been observed to increase from <2% to >37% (Gastrich et al., 2004). One such isolated virus, Aureococcus anophagefferns Virus (AaV), belongs to the Mimiviridae family (Moniruzzaman et al., 2014), in the realm Varidnaviria (ICTV, 2020). It is a large, icosahedral virus (Rowe et al., 2008), matching those visualized over the past few decades in natural blooms (Sieburth et al., 1988; Gastrich et al., 2004), and its genome encodes 377 putative coding sequences and 8 tRNAs (Moniruzzaman et al., 2014). Both PCR-based amplicon screening (Moniruzzaman et al., 2016) and transcriptomic analyses (Moniruzzaman et al., 2017) of brown tide bloom events have found signatures of viruses similar to AaV, suggesting the environmental relevance of this system.

To examine how viruses shape microbial community functions, researchers have taken advantage of metatranscriptomes of the cellular community to examine associated viruses (Moniruzzaman et al., 2017; Pound et al., 2020). Yet, RNA preparations from the environment are dominated by ribosomal RNA (rRNA) sequences (Urich et al., 2008). To reduce this signal and to increase the signal of the function-bearing messenger RNA (mRNA) sequences, researchers can decrease the rRNA component of a sample through “rRNA reduction.” Removal of rRNA in prokaryotic and mixed communities from the total RNA pool is generally completed through hybridization approaches of DNA oligonucleotides using different commercially available kits, with varying degrees of success (Petrova et al., 2017), or more recently, individual laboratory-designed oligonucleotides (Culviner et al., 2020). Historically, this process is thought to increase the retrieval of mRNA sequences from the plankton community (van Vliet, 2010). In cases where eukaryotes are of interest, researchers can take advantage of the long polyadenylation (poly-A) “tails” on transcripts, as during the processing and maturation of mRNAs a poly-A tail is added (Eckmann et al., 2011), and use the approach colloquially known as poly-A selection. This approach selects primarily for eukaryotic mRNA transcripts and has been reasoned to also select for viruses infecting eukaryotic algae (Blanc et al., 2014; Rosenwasser et al., 2014; Moniruzzaman et al., 2017). Yet, to our knowledge, there have been no direct comparisons of how these two approaches enrich for viral RNA (either transcripts or genomes) and whether these enrichments are reflective of the actual viral communities, in environmental samples. The goal of the current study was to leverage existing knowledge of virus–host dynamics in brown tide blooms to assess how sample preprocessing before RNA sequencing influenced analyses of entire viral communities within metatranscriptomes. RNA samples from two locations, Quantuck Bay and Tiana Beach, Long Island, NY, United States, were collected over the progression of a 10-week brown tide. RNA samples from each sampling event were processed in two ways prior to sequencing: poly-A selection or rRNA reduction, allowing for a paired comparison. The results provided an opportunity to both examine the development of the viral community during a bloom event: more strikingly, the observations contrast these disparate approaches and demonstrate how sample preparation shapes the quantitative ecological determination of viral effects in marine surface waters.

## Meterials and Methods

### Sampling and Sequencing

Samples were collected from brown tide blooms that occurred in Quantuck Bay (40°48′11.1″ N, 72°37′12.7″ W) and Tiana Beach (40°49′43.0″ N, 72°31′54.5″ W) from early June to mid-August in 2016. Whole water was preserved with glutaraldehyde (1% final vol/vol), stored at 4°C, and analyzed using a monoclonal antibody *via* an immunofluorescent flow cytometric technique (Stauffer et al., 2008) to obtain *A. anophagefferens* concentrations. Phycocyanin-containing (PC) cyanobacteria, phycoerythrin-containing (PE) cyanobacteria, and pico-eukaryotes were quantified *via* flow cytometry with a CytoFLEX Flow Cytometer (Beckman Coulter Life Sciences, Indianapolis, IN, United States) based on relative levels of chlorophyll *a* (Chl *a*) and phycoerythrin content. PC cyanobacteria were characterized by low content of phycoerythrin compared to PE cyanobacteria, which contain a high PE–to–Chl *a* ratio. Picoeukaryotes were distinguished by their relatively small size, the presence of high Chl *a*, and absence of phycoerythrin (Kang et al., 2017). Environmental RNA samples were collected by filtering approximately 25 mL of seawater onto replicate 47-mm filters and then flash frozen in liquid nitrogen within minutes on site. This volume of water has previously been used for a transcriptomic-based approach to study the dynamics of *A. anophagefferens* (Wurch et al., 2019) and viral communities (Moniruzzaman et al., 2017) in brown tides. The RNA samples that were to be preprocessed by rRNA reduction were collected on 0.2-μm polycarbonate filters, whereas those to be preprocessed by poly-A selection were collected on 1.0-μm polycarbonate filters. Frozen filters were maintained at −80°C before processing. CTAB buffer (Teknova, Hollister, CA, United States) and 1% mass/volume polyvinylpyrrolidone were added to each sample before RNA extractions. RNA extractions were performed using the UltraClean Plant RNA Isolation Kit (Qiagen, Hilden, Germany), using the modified manufacturer’s instructions for extractions with CTAB. To remove DNA, samples were treated with TURBO DNase (Ambion, Austin, TX, United States). RNA was quantified spectrophotometrically for yield and purity using an Agilent Bioanalyzer System (Agilent, Santa Clara, CA, United States). RNA samples then went through poly-A selection using a TruSeq Stranded mRNA Library Prep selection with oligo-dT beads (Illumina, San Diego, CA, United States) or rRNA reduction using a Ribo-Zero Gold kit (Illumina). After RNA processing, samples were sequenced using an Illumina HiSeq 2000 (Illumina) at the Columbia Genome Center (New York, NY, United States).

### Library Processing and Read Mappings to Reference Genomes

Reads were trimmed for quality using default parameters in CLC Genomic Workbench version 12 (Qiagen) (Supplementary Table 1). Trimmed reads were mapped to the coding sequences of the *A. anophagefferens* CCMP1984 (Gobler et al., 2011), AaV (Moniruzzaman et al., 2017), and *Synechococcus* species WH8101 (Marston and Polson, 2020) genomes. This *Synechococcus* species was chosen as its genome had the most top BLASTx hits within the genus to assembled contigs (see below) when translated contig sequences were queried against all cyanobacterial proteins (DIAMOND BLASTx version 0.9.31.132) (Buchfink et al., 2015). Reads were mapped using a 90% similarity fraction over a 90% length fraction in CLC Genomics Workbench version 12.0 (Qiagen). These parameters have been used previously for analysis of viral communities in aquatic metatranscriptomes (Pound et al., 2020). Trimmed reads from eight poly-A selected transcriptomic libraries collected during a 2011 brown tide bloom in Quantuck Bay (Moniruzzaman et al., 2017; Wurch et al., 2019) were also mapped to the AaV genome using the same parameters as above (Supplementary Table 2). All reads from a single time point (6/27/2016) were classified using the Kaiju online web server using default parameters (Menzel et al., 2016).

### Read Mappings to Viral Contigs

Contigs were assembled from trimmed reads of each library using MEGAHIT version 1.0.2 (Li et al., 2015). To find contigs that were putatively viral, a BLASTx-based approach was used that has been used previously for marker gene analyses of natural viral communities (Moniruzzaman et al., 2017; Pound et al., 2020). All viral protein sequences and taxonomic information was downloaded from NCBI in July 2020 (Brister et al., 2015). All translated contigs were queried against all viral proteins (DIAMOND version 0.9.31.132) (Buchfink et al., 2015). Contigs with BLASTx hits (*e*-value cutoff < 1 × 10^–10^) were retained. To remove contigs originating from cellular organisms, translated retained contigs were queried against the non-redundant database (download May 2020) (NCBI Resource Coordinators, 2016) using DIAMOND version 0.9.31.132 (Buchfink et al., 2015). Contigs with top BLASTx hits (*e*-value cutoff < 1 × 10^–10^) that were cellular in origin were removed. These contigs were removed by screening top hits against all viral proteins using a python script; if the top hit was found within the database of all viral proteins, the contigs were retained, and if not, they were removed. The remaining contigs were considered viral for this analysis. To assign phylogeny, translated contigs were queried against all viral proteins using BLAST version 2.10.0 + (Camacho et al., 2009). Taxonomic information from the top BLASTx hit for each contig was used to assign viral realm (ICTV, 2020), family, and organism (Supplementary Data 1). Coding sequences were excised from full-length contigs as described previously (Gann et al., 2019; Pound et al., 2020).

As the majority of members of the realm Riboviria have RNA genomes, it was determined whether any of these contigs were majority complete as described previously (Moniruzzaman et al., 2017; Pound et al., 2020). In this study, we define these contigs to be majority complete if both the RNA-dependent RNA polymerase (RDRP) and a structural protein were present on the contig. To identify majority complete contigs, all translated Riboviria contigs were queried against all Riboviria proteins within RefSeq (downloaded in November 2020) using BLAST version 2.10.0 + (Camacho et al., 2009). Contigs with multiple unique aligned portions were excised as described above, and the translated sequences were queried against all Riboviria proteins using BLAST version 2.10.0 + (*e*-value cutoff < 1 × 10^–10^) (Camacho et al., 2009). A Pfam domain search (Pfam database v32) was performed for the top BLASTx hit for each aligned portion of the contig using CLC Genomics Workbench version 12.0 (Qiagen) (El-Gebali et al., 2019). If domains for both a structural protein (capsid or coat protein) and the RDRP were present, the contig was considered majority complete.

Redundancies were removed by clustering coding sequences using CD-HIT-EST version 4.7 with a 0.90 sequence identity threshold (Fu et al., 2012). Trimmed reads from the library each set of contigs was assembled from were mapped to those contigs as described above. To compare contigs generated from the poly-A selected libraries and the rRNA-reduced libraries, a BLASTn was performed (BLAST version 2.10.0 +) (Camacho et al., 2009). Contigs were considered to be in both types of libraries only if reciprocal top BLASTn hits (*e*-value cutoff < 1 × 10^–10^) were present. Paired *t*-tests for comparisons between poly-A selected libraries and rRNA-reduced libraries, as well as regressions, were performed in Prism version 8.4.3 (GraphPad, San Diego, CA, United States).

## Results

### Description of Sampling and RNA Sequencing

**FIGURE 1:**
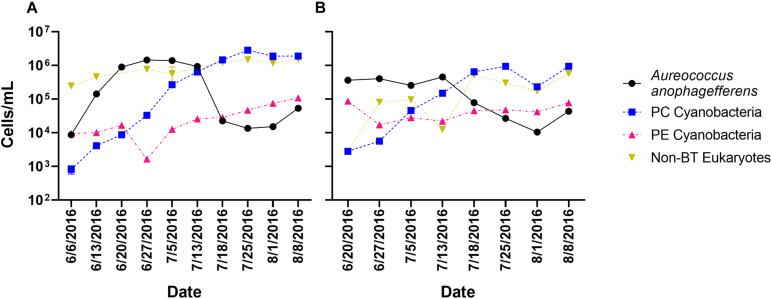
Cell concentrations over the sampling period in **(A)** Quantuck Bay and **(B)** Tiana Beach. PC Cyanobacteria: phycocyanin-containing cyanobacteria; PE Cyanobacteria: phycoerythrin-containing cyanobacteria; non-BT Eukaryotes: non–*A. anophagefferens* eukaryotes. Error bars represent ± SD and, where not visible, are within the size of the symbol.

Twenty-six time points had samples collected, processed, and sequenced from two locations (Quantuck Bay and Tiana Beach, NY) during a 2016 brown tide event. Sampling captured the entire bloom progression (initiation, peak, decline) over 10 weekly sampling points (with four time points with triplicate sampling) in Quantuck Bay (Figure 1A), whereas eight weekly sampling points in Tiana Beach captured the peak and collapse of the bloom (Figure 1B). In both locations, the collapse of the *A. anophagefferens* bloom was followed by a bloom in phycocyanin-containing cyanobacteria (Figures 1A,B). RNA from each of the 26 sampling points was sequenced after either poly-A selection or rRNA-reduction, generating 52 transcriptomic libraries with approximately 4.41 billion quality control trimmed reads (Supplementary Table 1).

### Read Mappings to *A. anophagefferens* and AaV Showed Different Trends Based on Library Type

**FIGURE 2:**
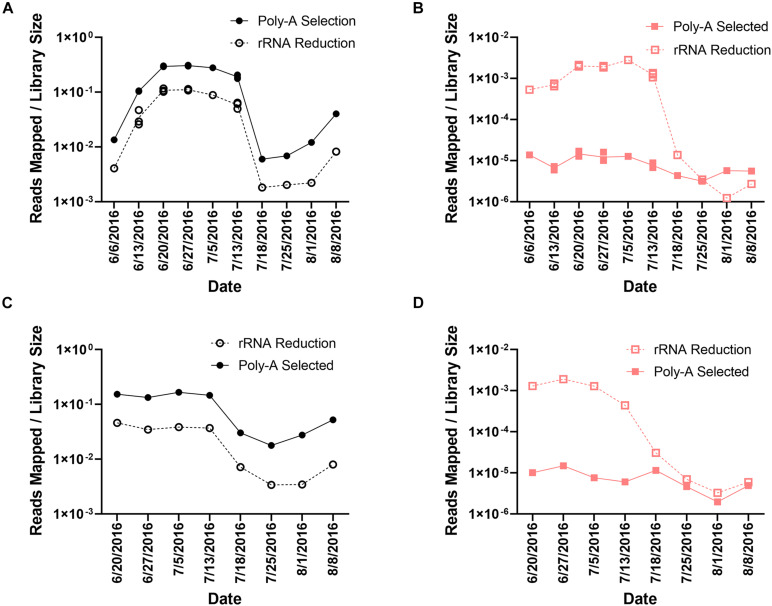
Read mappings to *A. anophagefferens* and AaV. Library normalized reads mapping to **(A)** *A. anophagefferens* and **(B)** AaV in Quantuck Bay, and library normalized reads mapping to **(C)** *A. anophagefferens* and **(D)** AaV in Tiana Beach. On dates where multiple libraries exist, all individual values are plotted.

In the poly-A selected libraries, the percentage of reads mapping to *A. anophagefferens* CCMP1984 ranged from 0.60% (8.94 × 10^5^ reads) to 30.16% (3.92 × 10^7^ reads) (Supplementary Table 1 and Figures 2A,C) of the total library. The number of reads mapping to *A. anophagefferens* was less in the rRNA-reduced samples, ranging from 0.18% (9.63 × 10^4^ reads) to 11.70% (7.04 × 10^6^ reads) of the total library (Supplementary Table 1 and Figures 2A,C). Even though the proportion of the library that mapped to the *A. anophagefferens* CCMP1984 reference genome was significantly different (paired *t*-test, *p* < 0.0001) between paired libraries from each sample, there was a strong correlation with the proportion of the library mapped to the *A. anophagefferens* genome based on library preparation at a given sample, in both Quantuck Bay (*R*^2^ = 0.9752) and Tiana Beach (*R*^2^ = 0.9653) (Supplementary Figure 1A). To further support that differences seen with the read mappings to *A. anophagefferens* were due to RNA processing, all reads were taxonomically classified from a single time point at peak bloom (June 27, 2016) (Supplementary Table 3). As with the read mappings to the *A. anophagefferens* reference genome, there was a higher proportion of reads that were identified as belonging to the dominant alga in the poly-A selected samples compared to the rRNA-reduced samples. Also, almost all (38/42) the abundant taxa in the bloom (> 0.1% of identified reads classified to that taxa) had significantly (paired *t*-test, *p* < 0.05) different proportions of the reads identified to that taxa (Supplementary Table 3).

These trends did not emerge from read mappings to the AaV genome. In total, 1.52 × 10^6^ reads mapped to AaV in the rRNA-reduced libraries, whereas only 2.6 × 10^4^ reads mapped in the poly-A selected libraries. The reads in the poly-A selected samples represented only 1.73% of the number of reads mapped in the rRNA-reduced libraries. In the poly-A selected libraries, the percentage of reads mapped to the AaV genome ranged from 1.97 × 10^–4^% (150 reads) to 1.69 × 10^–3^% (1.64 × 10^3^ reads) (Supplementary Table 1 and Figures 2B,D). The range was much greater in the rRNA-reduced libraries, ranging from 1.23 × 10^–4^% (69 reads) to 0.28% (1.51 × 10^5^ reads) of the total reads in the library (Supplementary Table 1 and Figures 2B,D). The proportion of the reads that mapped to AaV also significantly differed (paired *t*-test, *p* < 0.0001) between library pairs. There was a weaker correlation between the proportion of the library mapping to the AaV genome based on library preparation at a given sample in both Quantuck Bay (*R*^2^ = 0.5618) and Tiana Beach (*R*^2^ = 0.5062) relative to the correlation observed for *A. anophagefferens* in the two library pairs (Supplementary Figure 1B). Comparing reads mapping to AaV to those mapping to *A. anophagefferens* showed that there was a stronger correlation in the rRNA-reduced libraries compared to the poly-A selected libraries in both Quantuck Bay (rRNA reduced: *R*^2^ = 0.8585; poly-A selected: *R*^2^ = 0.5006) and Tiana Beach (rRNA reduced: *R*^2^ = 0.6900; poly-A selected: *R*^2^ = 0.1892) (Supplementary Figures 2A,B). Trimmed reads from eight poly-A selected transcriptomic libraries from 3 days during a 2011 bloom in Quantuck Bay, NY (Moniruzzaman et al., 2017; Wurch et al., 2019), were also mapped to the AaV genome using the same parameters (Supplementary Table 2). Like the poly-A selected libraries from this study, a small percentage of the total libraries mapped to the AaV genome: ranging from 6.74 × 10^–4^% (644 reads) to 4.03 × 10^–3^% (1.76 × 10^3^ reads).

### Viral Contig Assembly and Read Mapping Were Influenced by Library Type

**FIGURE 3:**
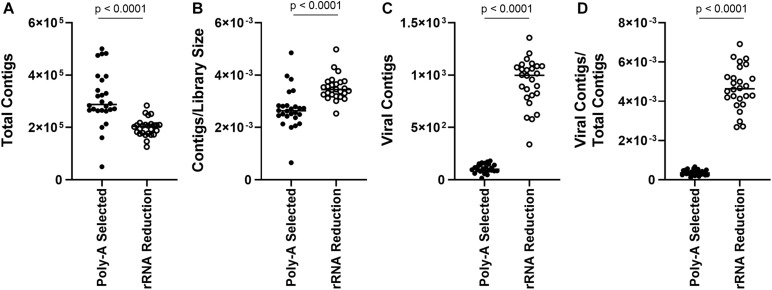
Comparison of contigs between poly-A selected and rRNA-reduced libraries. **(A)** Total contigs, **(B)** contigs normalized to library size, **(C)** total viral contigs, and **(D)** viral contigs normalized to total contigs between the two types of libraries.

As reads mapping to the AaV genome were influenced by preprocessing of the sample, we assessed how representation of other members of the viral community were influenced. Overall, there were more total contigs assembled in the poly-A selected libraries compared to the rRNA-reduced libraries (paired *t*-test, *p* < 0.0001) (Figure 3A), although rRNA-reduced libraries had more contigs when the data were normalized by library size (paired *t*-test, *p* < 0.0001) (Figure 3B). A total of 2.70 × 10^4^ contigs of predicted viral origin were identified and denoted as “viral contigs” using a BLASTx-based approach. There were significantly more viral contigs (paired *t*-test, *p* < 0.0001) and viral contigs per total contigs (paired *t*-test, *p* < 0.0001) in the rRNA-reduced libraries (Figures 3C,D). In total, there were 2.80 × 10^3^ viral contigs assembled from the poly-A selected libraries; only 11.54% of the 2.42 × 10^4^ viral contigs assembled from the rRNA-reduced libraries. In the rRNA-reduced libraries, there was an average of 3.12 × 10^5^ total reads mapped (SD ± 1.41 × 10^5^ reads) to viral contigs in Quantuck Bay (Supplementary Figure 3A), and 1.81 × 10^5^ total reads mapped (SD ± 1.46 × 10^5^ reads) in Tiana Beach (Supplementary Figure 3C), making up an average 0.53 and 0.33% of the total reads in the libraries, respectively. Far fewer reads mapped to viral contigs in the poly-A selected libraries with 2.60 × 10^4^ total reads (SD ± 1.81 × 10^4^ reads) and 2.22 × 10^4^ total reads (SD ± 1.54 × 10^4^ reads) mapped to viral contigs in Quantuck Bay (Supplementary Figure 3A) and Tiana Beach (Supplementary Figure 3C), respectively. This made up only 0.023 and 0.020% of the total reads, on average, in the libraries. When reads mapped were normalized to library size (Supplementary Figures 3B,D), there were significantly fewer reads mapping to viral contigs in the poly-A selected libraries (paired *t*-test, *p* < 0.0001). The number of reads mapped based on sample preprocessing did not strongly correlate with one another in samples from either Quantuck Bay (*R*^2^ = 0.3242) or Tiana Beach (*R*^2^ = 0.5164) (Supplementary Figure 4).

**FIGURE 4:**
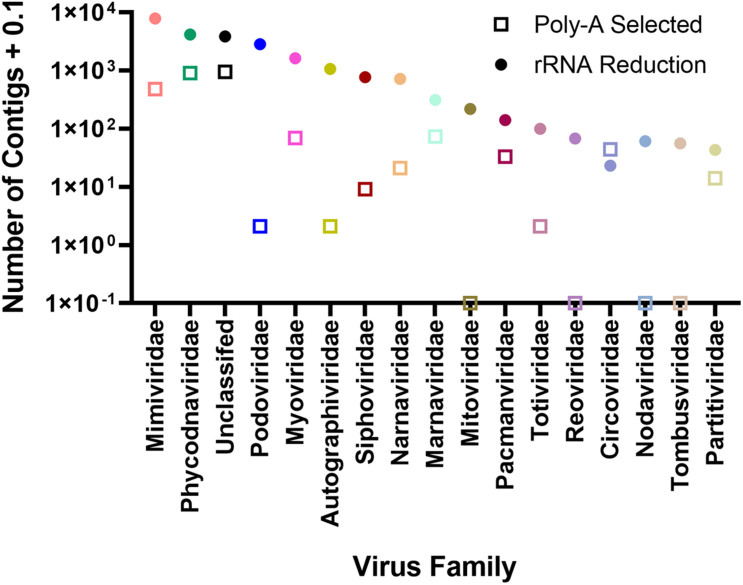
Number of contigs assigned to viral family separated by whether contigs were assembled from rRNA-reduced libraries or poly-A selected libraries.

To assign phylogenies to viral contigs, taxonomic information from the top BLASTx hit was used. To validate this approach, we compared reads mapping to the AaV reference genome with those contigs with a top BLASTx hit to the genome (Supplementary Figure 5). In both sampling locations, the two correlated well (Quantuck Bay: *R*^2^ = 0.7526; Tiana Beach: *R*^2^ = 0.8398) in the rRNA-reduced libraries. Although all contigs have an assigned phylogeny (Supplementary Data 1), only those that make up > 0.1% the total contigs will be further discussed. There were more unique contigs in all viral realms in the rRNA-reduced libraries, except for the Monodnaviria (ssDNA viruses, Supplementary Table 4). In both poly-A selected and rRNA-reduced libraries, more than half of the contigs were grouped as Varidnaviria (tailless dsDNA viruses; 53.49 and 50.68%, respectively). For contigs assembled from poly-A selected libraries, the only other realm that made up a large portion (>5%) of the contigs was the Riboviria (RdRp and RdDP encoding viruses; 39.51%). In the rRNA-reduced libraries, both Duplodnaviria (tailed phage; 26.23%) and Riboviria (21.95%) made up a large portion of the total viral contigs assembled from those libraries (Supplementary Table 4). It is worth noting that there appear to be several near-complete Riboviria members assembled during this study as has been seen by others (Moniruzzaman et al., 2017; Pound et al., 2020). Of the 6.41 × 10^3^ contigs grouped as Riboviria, 158 were considered majority complete, containing both Pfam domains for a structural protein and an RDRP (Supplementary Table 5). Of the 1.09 × 10^3^ Riboviria contigs assembled from poly-A selected libraries, 48 (4.39%) were majority complete, whereas 110 (2.07%) of 5.31 × 10^3^ contigs assembled from rRNA-reduced libraries were majority complete. The families with the most viral contigs assembled from the poly-A selected libraries were unclassified (35.54%), Phycodnaviridae (33.68%), and Mimiviridae (17.89%). The remaining families made up < 5% of the total viral contigs. The distribution of viral contigs assembled from rRNA-reduced libraries by family differed from the poly-A selected libraries, with a greater representation of more families: Mimiviridae (32.67%), Phycodnaviridae (17.26%), unclassified (15.98%), Podoviridae (11.84%), and Myoviridae (6.78%) (Figure 4 and Supplementary Table 6). It should be noted that many families of viruses are not found in this dataset using poly-A selection including several RNA virus families (Tombusviridae, Nodaviridae, Reoviridae, and Mitoviridae) but are found in the rRNA-reduced library dataset (Supplementary Table 6). Others (Podoviridae, Autographiviridae, Siphoviridae, Totiviridae) have fewer than 10 contigs (Supplementary Table 6). To determine if any contigs were similar in both poly-A selected and rRNA-reduced libraries, contigs from each library type were compared by BLASTn. Those that had reciprocal top BLASTn hits were considered present in both. Only 596 contigs of 2.80 × 10^3^ viral contigs assembled from the poly-A selected libraries were found to be shared in the 2.42 × 10^4^ viral contigs assembled from the rRNA-reduced libraries. The majority of these contigs were in the families Phycodnaviridae (41.28%), unclassified (24.16%), and Mimiviridae (20.47%) (Supplementary Table 7).

### Richness and Diversity of Viral Contig Phylogeny Were Influenced by Library Type

**FIGURE 5:**
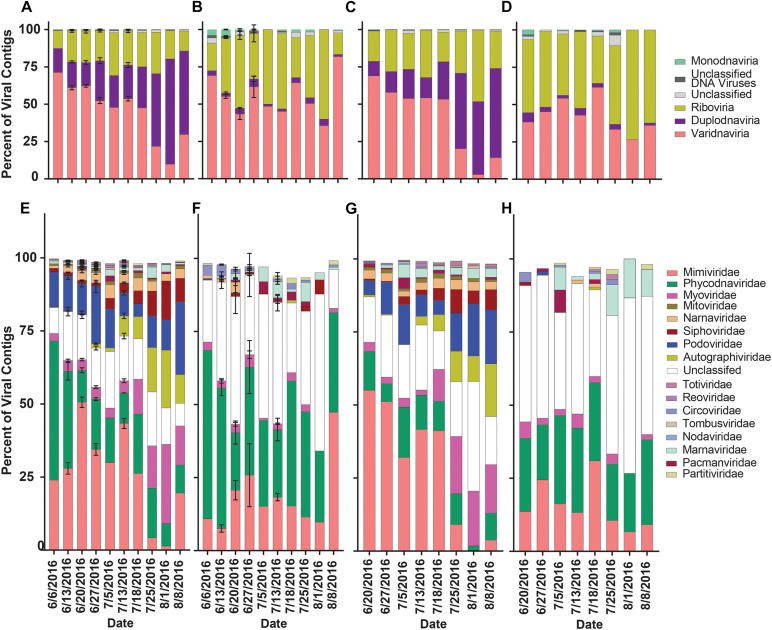
Relative proportion of viral contigs by phylogeny, date and sampling location. Proportions of viral contigs from **(A)** rRNA-reduced and **(B)** poly-A selected Quantuck Bay libraries grouped by realm. Proportions of viral contigs from **(C)** rRNA-reduced and **(D)** poly-A selected Tiana Beach libraries grouped by realm. Proportions of viral contigs from **(E)** rRNA-reduced and **(F)** poly-A selected Quantuck Bay libraries grouped by family. Proportions of viral contigs from **(G)** rRNA-reduced and **(H)** poly-A selected Tiana Beach libraries grouped by family. On dates where multiple samples exist, standard deviation is shown by error bars.

To assess how library preparation influenced the overall richness and diversity of viral contigs in this dataset, contigs were separated by library. Reads from the library were mapped to aligned portions of viral contigs. Contigs were then clustered by realm and family to assess richness (proportion of viral contigs) and diversity (reads mapped to viral contigs). In the rRNA-reduced libraries from both Quantuck Bay (Figure 5A) and Tiana Beach (Figure 5C), contigs from Varidnaviria made up the largest proportion of viral contigs before bloom collapse and then were succeeded by contigs from Duplodnaviria. The abundance of contigs in other realms remained consistent. These trends were not seen in the poly-A selected libraries, where there appeared to be little change in the proportion of contigs grouped by realm, with contigs from Varidnaviria and Riboviria making up the overwhelming majority in both sampling locations (Figures 5B,D). These trends appeared when clustering viral contigs by family as well. In the rRNA-reduced samples, there was a reduction in the proportion of contigs in viral families that infect eukaryotes (Phycodnaviridae and Mimiviridae) and an increased proportion in those infecting prokaryotes (Myoviridae, Podoviridae, Siphoviridae, Autographiviridae), in both locations (Figures 5E,G), upon bloom collapse. Changes in the proportion of contigs were not observed in the poly-A selected libraries (Figures 5F,H). There were significant (paired *t*-test, *p* < 0.05) differences in the proportions of almost all families (16/17) when comparing poly-A selected and rRNA-reduced libraries by sample (Supplementary Table 8).

**FIGURE 6:**
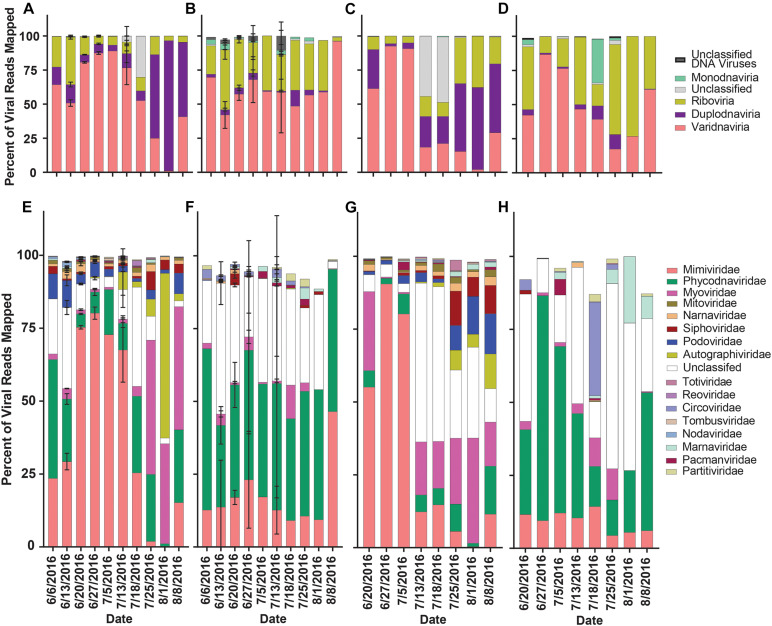
Relative proportion of reads mapped to viral contigs by phylogeny by date and sampling location. Proportions of reads mapped to viral contigs from **(A)** rRNA-reduced and **(B)** poly-A selected Quantuck Bay libraries by realm. Proportions of viral contigs from **(C)** rRNA-reduced and **(D)** poly-A selected Tiana Beach libraries by realm. Proportions of read mapped to viral contigs from **(E)** rRNA-reduced and **(F)** poly-A selected Quantuck Bay libraries by family. Proportions of viral contigs from **(G)** rRNA-reduced and **(H)** poly-A selected Tiana Beach libraries by family. On dates where multiple samples exist, standard deviation is shown by error bars.

The proportion of normalized reads mapping to viral contigs (our proxy for diversity) grouped by realm in rRNA-reduced libraries again showed a transition from read mappings dominated by Varidnaviria members to Duplodnaviria members in both locations when the bloom collapsed (Figures 6A,C). In the poly-A selected libraries, no shift in proportions of read mappings was seen in Quantuck Bay (Figure 6B), while a shift from Varidnaviria members to Riboviria members was observed during bloom collapse in Tiana Beach (Figure 6D). When grouping contigs by family, in rRNA-reduced libraries, there was a shift from read mappings dominated by Mimiviridae members to those dominated by families infecting prokaryotes in both locations (Figures 6E,G). In contrast, the family with the largest proportion of reads mapping to it in the poly-A selected libraries was Phycodnaviridae (Figures 6F,H). There were significant (paired *t*-test, *p* < 0.05) differences in the proportions of read mappings to most families (11/17) when comparing read mappings of poly-A selected and rRNA-reduced libraries by sample (Supplementary Table 8). To further assess differences in the proportion of read mappings when contigs were grouped by family, normalized reads were mapped to the six viral families that represented the majority (>99.7%) of reads mapped (Supplementary Figure 6). For contigs in families that infect prokaryotes (Podoviridae, Autographiviridae, Myoviridae, Siphoviridae), the number of reads mapped in the poly-A selected libraries was a small percentage (<6%) of those mapped in the rRNA-reduced libraries per sample (Supplementary Figures 6C,D and Supplementary Table 9). This trend was not present for Mimiviridae and Phycodnaviridae members. Although both, on average, had fewer reads mapped to the poly-A selected libraries compared to their rRNA-reduced counterparts, Mimiviridae contigs (Quantuck Bay average: 5.39%, *SD* = 8.87; Tiana Beach: average = 12.67%, *SD* = 24.90) were more influenced by the library type than Phycodnaviridae (Quantuck Bay: average: 20.97%, *SD* = 37.33; Tiana Beach: average = 85.95%, *SD* = 148.05) (Supplementary Figure 6 and Supplementary Table 9).

## Discussion

Understanding the constraints of modern molecular tools when they are deployed to analyze microbial communities is a key facet of microbial ecology. With much of the current focus of this research being on pipelines and programs, it is sometimes easy to overlook the limitations of the techniques and chemistries used to collect sequencing data. The goal of the current study was to understand how preprocessing samples (i.e., poly-A selection *vs*. rRNA reduction) to be subjected to RNA sequencing influenced subsequent analyses of native viral community ecology. It has been shown in clinical samples differences in expression profile results based on the method used to enrich for mRNAs occurs (Zhao et al., 2018). We found that the rRNA reduction approach produced outcomes consistent with our understanding of ecosystem ecology, while the poly-A selected libraries did not. We frame these observations within the context of developing a more quantitative understanding of virus–host interactions in aquati c environments.

As many viruses that infect eukaryotes have been shown experimentally to have poly-A tails (Broyles, 2003; Byrne et al., 2009; Priet et al., 2015), their mRNA should be pulled down during poly-A selection in a manner similar to their eukaryotic host. For detection of active infections of viruses with DNA genomes (viral mRNAs) and RNA viruses in diverse environments, poly-A selection has been used (Levin et al., 2017; Moniruzzaman et al., 2017), as has sequencing total RNA (Correa et al., 2013; Nagano et al., 2019). In the laboratory, changes in the transcriptomic profiles of infected protists over the infection cycle have both been poly-A selected (Legendre et al., 2010; Blanc et al., 2014; Rosenwasser et al., 2014; Moniruzzaman et al., 2018) and rRNA-reduced (Rodrigues et al., 2020). Taking advantage of paired field samples processed by two differing approaches, coupled to a known virus–host system in brown tide bloom events, provided unique insight regarding how preprocessing of RNA influences the interpretation of the viral community from metatranscriptomes.

In our efforts, reads mapping to the *A. anophagefferens* and AaV genomes showed the AaV signal strongly correlated to its host, but only in the rRNA-reduced libraries. Unlike reads mapping to *A. anophagefferens*, the number of reads mapped to AaV by sample did not strongly correlate when comparing library type. The number of reads mapped to AaV in the rRNA-reduced libraries was also at least an order of magnitude higher compared to their poly-A selected pairs. This was not expected, as other Mimiviridae members (Mimivirus and *Megavirus chilensis*) have been shown to have poly-A tails on their mRNA (Byrne et al., 2009; Priet et al., 2015), and late in an infection cycle, approximately 15% of poly-A selected transcriptomic libraries mapped to the AaV genome in xenic algal cultures (Moniruzzaman et al., 2018). The low number of reads mapping to the AaV genome in this study, although not expected, was similar to the number of reads mapped in poly-A selected libraries during the peak and collapse of a brown tide in 2011 (Moniruzzaman et al., 2017). Taken together, these comparisons suggest that caution is warranted in the interpretation of infection dynamics of AaV from poly-A selected libraries.

The trend described above, of a bias against viral sequences in poly-A selected libraries, was not unique to AaV and indeed seemed to be common, if not universal across viral realms. There was an order of magnitude fewer viral contigs assembled and number of reads mapped to those viral contigs in the poly-A selected libraries compared to their rRNA-reduced pairs. All viral realms had fewer contigs assembled and reads mapped in the poly-A selected libraries, except for Monodnaviria, which generally had few contigs assemble (49 v. 31 total), and as such, the observation is likely a function of sampling depth, not richness. Interestingly, the Mimiviridae and Phycodnaviridae families (both within Varidnaviria) were influenced differently by the library preparation. In rRNA-reduced libraries, there were nearly twice the number of Mimiviridae contigs as Phycodnaviridae contigs assembled, whereas in poly-A selected libraries, the reverse was observed. This trend was also seen in the reads mapped to the contigs. It is unclear why the difference between the two viral families exists, as both infect protists (Wilhelm et al., 2017). It is possible that there is a difference in mRNA structure, as it has been shown that secondary structures in nucleotides can reduce hybridization efficiency (Koehler and Peyret, 2005), so it is possible that as a large portion of the poly-A tails in some Mimiviridae members form hairpins (Byrne et al., 2009; Priet et al., 2015), poly-A selection may be biased against these mRNA. Understanding the mechanisms that drive differences in the ability of poly-A selection protocols to efficiently pull down various viral family transcripts is worth comparing in the future. Regardless, this comparison highlights the role of library type in calculations of viral community diversity and thus emphasizes that library type should be carefully targeted to the ecological question for field studies.

This work demonstrated a shift in abundance of virus signatures from viruses infecting eukaryotes to those infecting cyanobacteria after the bloom collapsed, but this was only able to be observed in rRNA-reduced libraries. Although it was unsurprising that changes in reads mappings to cyanophage/bacteriophage families could not be detected in poly-A selected libraries, as most of their bacterial hosts do not add long poly-A tails to their mRNA (van Vliet, 2010), the dynamics of families infecting eukaryotic viruses was a surprise. The rRNA-reduced library showed a known environmentally relevant host–virus system correlating with one another, and this family of viruses made up the largest proportion of contigs and mapped reads only while their host abundance was also high. In other algal blooms, virus concentrations follow those of their host (Tarutani et al., 2000), so this was to be expected. The poly-A selected libraries had only low levels of reads mapped to AaV, as well as an increased prevalence of reads mapping to Phycodnaviridae, a family of viruses not known to infect *A. anophagefferens*. An interpretation using only the poly-A selected metatranscriptomes would have given drastically different conclusions of the viral ecology in this system. We note that the filter size used to collect samples for RNA was different for the poly-A selected (1 μm) and rRNA-reduced (0.2 μm) libraries, as ours was an unforeseen observation that emerged from the data when looking at the *A. anophagefferens* AaV host–virus system read mappings. Differences in filter size could introduce some variance into our study, and so this study should be repeated with the same size filter to eliminate this bias. However, we believe the trends seen are mostly due to differences in RNA processing, not filter size. We note this study examined cell-associated viruses. Small viruses (such as RNA viruses and some phage) would pass through both filter types, whereas large DNA viruses may be collected on the 0.2-μm filters, yet their DNA genomes would not be included in these metatranscriptomic datasets. Therefore, this dataset should be enriched in active infections regardless of the filter size, and trends seen should reflect differences in RNA processing.

Although the data presented here suggest not using poly-A selected metatranscriptomes to understand viral dynamics in transcriptomes generated from natural communities, this does not say information cannot be gleaned from metatranscriptomes that were poly-A selected. First, if the primary research question is studying the eukaryote expression patterns in the environment, poly-A selected metatranscriptomes should be used because of the increased signal. Viral diversity studies can still be conducted with these, as many viral contigs from diverse families can still be assembled. Also, poly-A selected metatranscriptomes can be used for RNA viromes of aquatic systems, as there would not be the large signal from eukaryotic transcripts as typically these have the cellular-size fraction removed before extraction and sequencing. Finally, using poly-A selected transcriptomes can still be used in laboratory host–virus systems to understand infection cycle dynamics. For example, transcriptomes of cultures infected with Varidnaviria members were poly-A selected, and this allowed for a fundamental understanding of early and late virally encoded genes, as well as changes in host transcript expression patterns (Legendre et al., 2010; Blanc et al., 2014; Moniruzzaman et al., 2018).

While studying viral dynamics with poly-A selected transcriptomes may be appropriate in certain circumstances, the data herein suggest that careful consideration of the library type is warranted in future studies. Even though eukaryote-infecting viruses are not as efficiently detected at the community level in poly-A selected libraries compared to rRNA-reduced ones, the data can provide some insight into natural viral communities. As there appears to be a bias against viral sequences of all types in poly-A selected libraries, and rRNA-reduced metatranscriptomes produced outcomes more consistent with our view of brown tide bloom ecology, going forward using the rRNA-reduced metatranscriptome approach would be recommended for community-level studies. Our observations provide rationale for continued efforts to isolate new virus–host systems and a pressing need to validate methodology for each.

## Data Availability Statement

The datasets presented in this study can be found in online repositories. Raw sequencing data from this study is deposited to the Short Reads Archive under the Bioproject number PRJNA689205. Python scripts used in the analysis of the data are deposited to GitHub (https://github.com/Wilhelmlab/Gann2021-BtB-transcriptomes).

## Author Contributions

EG and SW designed the study. YK, CG, and SD designed and performed sampling and RNA process. EG performed the bioinformatic analyses. All authors contributed to the drafting of the manuscript.

## Conflict of Interest

The authors declare that the research was conducted in the absence of any commercial or financial relationships that could be construed as a potential conflict of interest.
