# Supplementary material for: Metatranscriptome Library Preparation Influences Analyses of Viral Community Activity During a Brown Tide Bloom
Source: Front Microbiol. 2021 May 31;12:664189. doi: 10.3389/fmicb.2021.664189 (PMC8200674; doi:10.3389/fmicb.2021.664189)
Supplement: Supplementary file 1 [file Data_Sheet_1.pdf]

Supplemental Information to be published with: Metatranscriptome library preparation influences analyses of viral community activity during a brown tide bloom

Eric R. Gann[1], Yoonja Kang[2], Sonya T. Dyhrman[3,4], Christopher J. Gobler[5], Steven W. Wilhelm[1*]

[1]University of Tennessee, Department of Microbiology, Knoxville, Tennessee, USA

[2]Chonnam National University, School of Marine Technology, Department of Ocean Integrated Science, Yeosu, South Korea

[3]Columbia University, Biology and Paleo Environment Division, Lamont-Doherty Earth Observatory, Palisades, New York, USA

[4]Columbia University, Department of Earth and Environmental Sciences, Palisades, New York, USA

[5]Stony Brook University, School of Marine and Atmospheric Sciences, Stony Brook University, New York, USA

*** Correspondence:**

Steven W. Wilhelm

wilhelm@utk.edu Telephone: 1-865-974-0665 Fax: 1-865-974-4007

Keywords: library preparation, rRNA reduction, polyadenylation selection, virus ecology

Supplemental Datasets Legends.

Supplemental Dataset 1. Description of viral contigs determined in this study.

Supplemental Figure 1. Correlations between samples based on processing of RNA prior to sequencing (poly-A selection v. rRNA reduction). Correlations between reads mapped to A) *A. anophagefferens* and B) AaV at both locations.

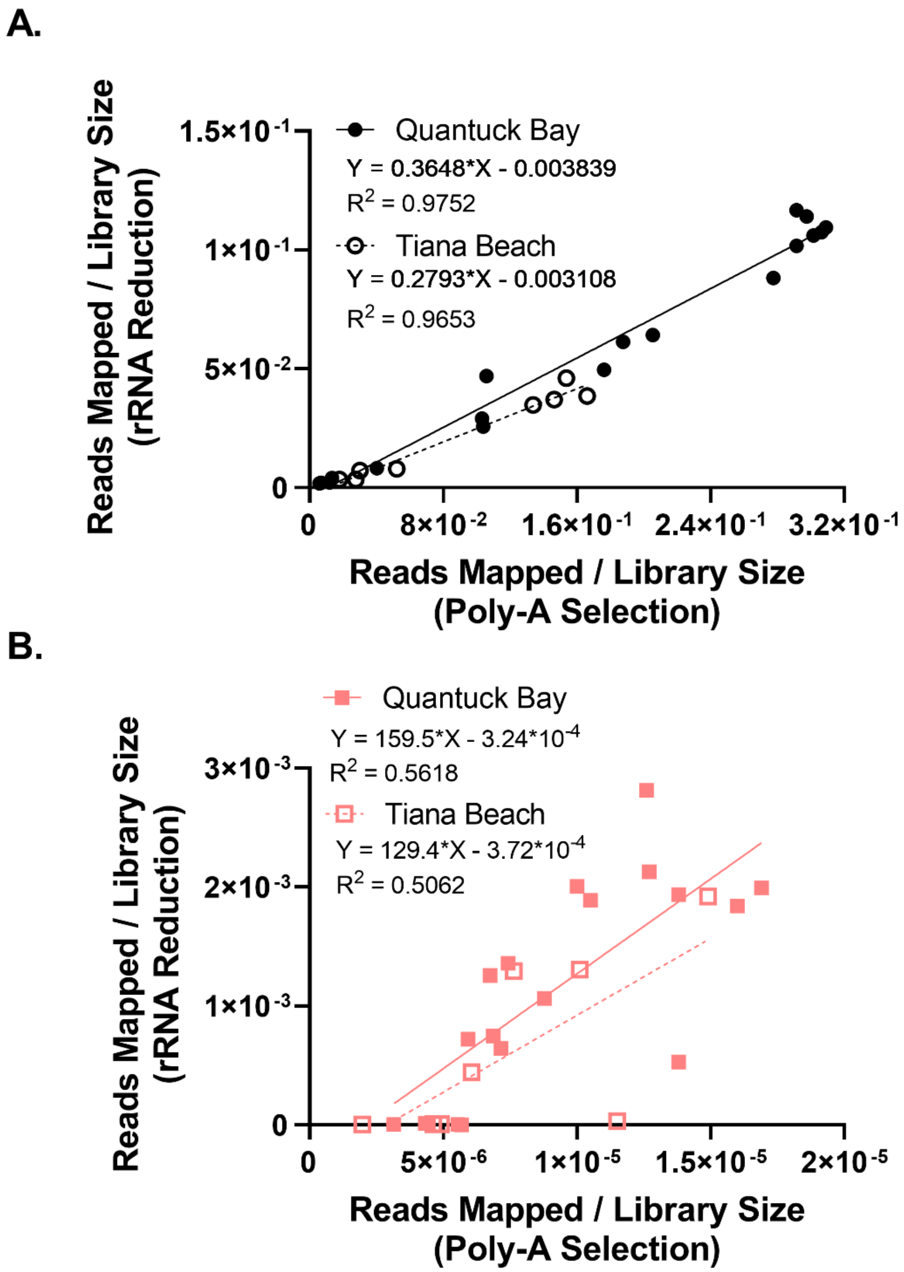

Supplemental Figure 2. Correlations between reads mapped to AaV v. *A. anophagefferens* in each sample. A) Correlations from samples where RNA was poly-A selected prior to sequencing. B) Correlations from samples were RNA was rRNA reduced prior to sequencing.

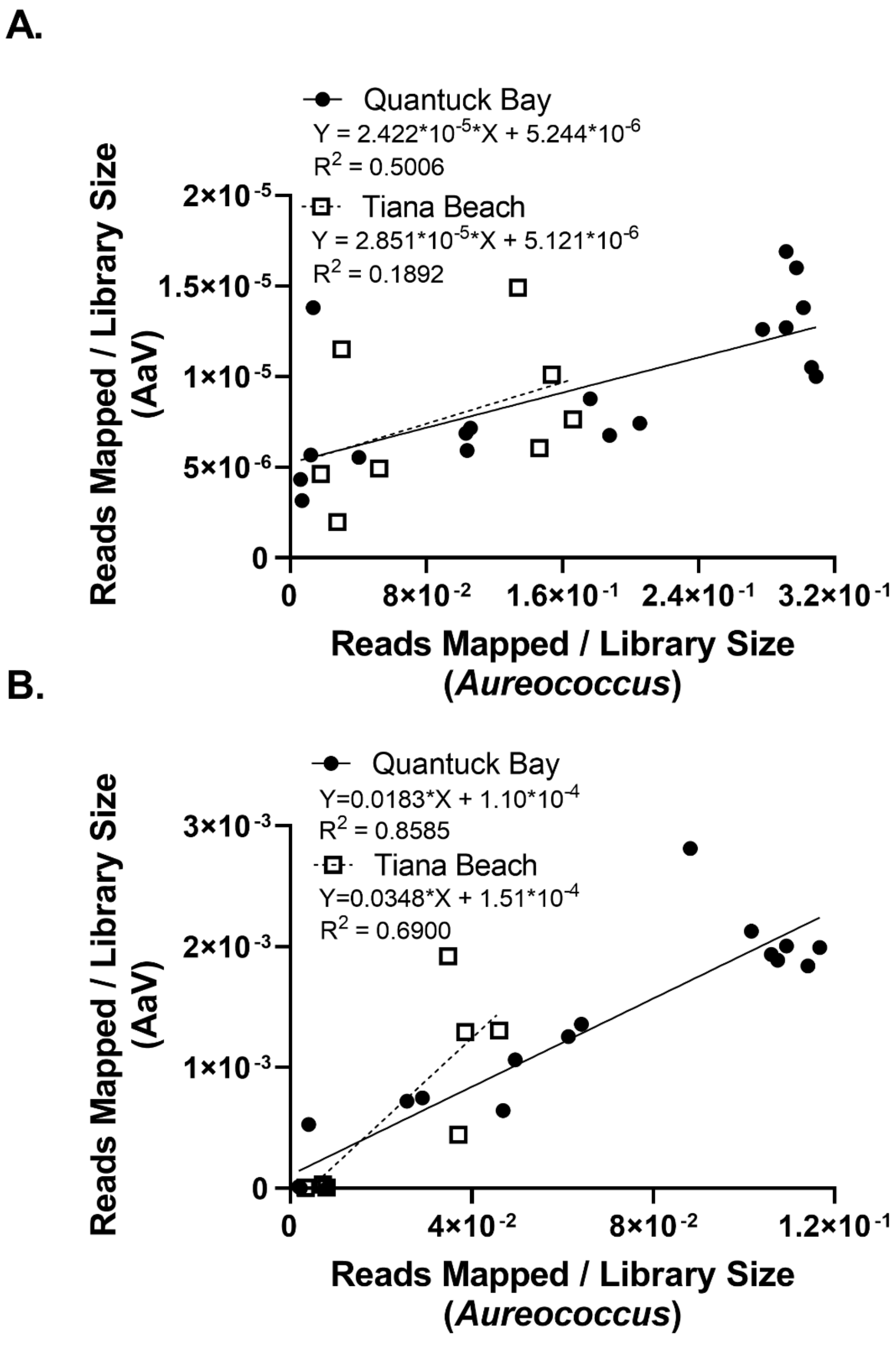

Supplemental Figure 3. Reads Mappings to Viral contigs. Total read mapping values to libraries in A) Quantuck Bay and C) Tiana Beach. Library normalized read mapping values in B) Quantuck Bay and D) Tiana Beach. On dates where multiple libraries exist, all individual values are plotted.

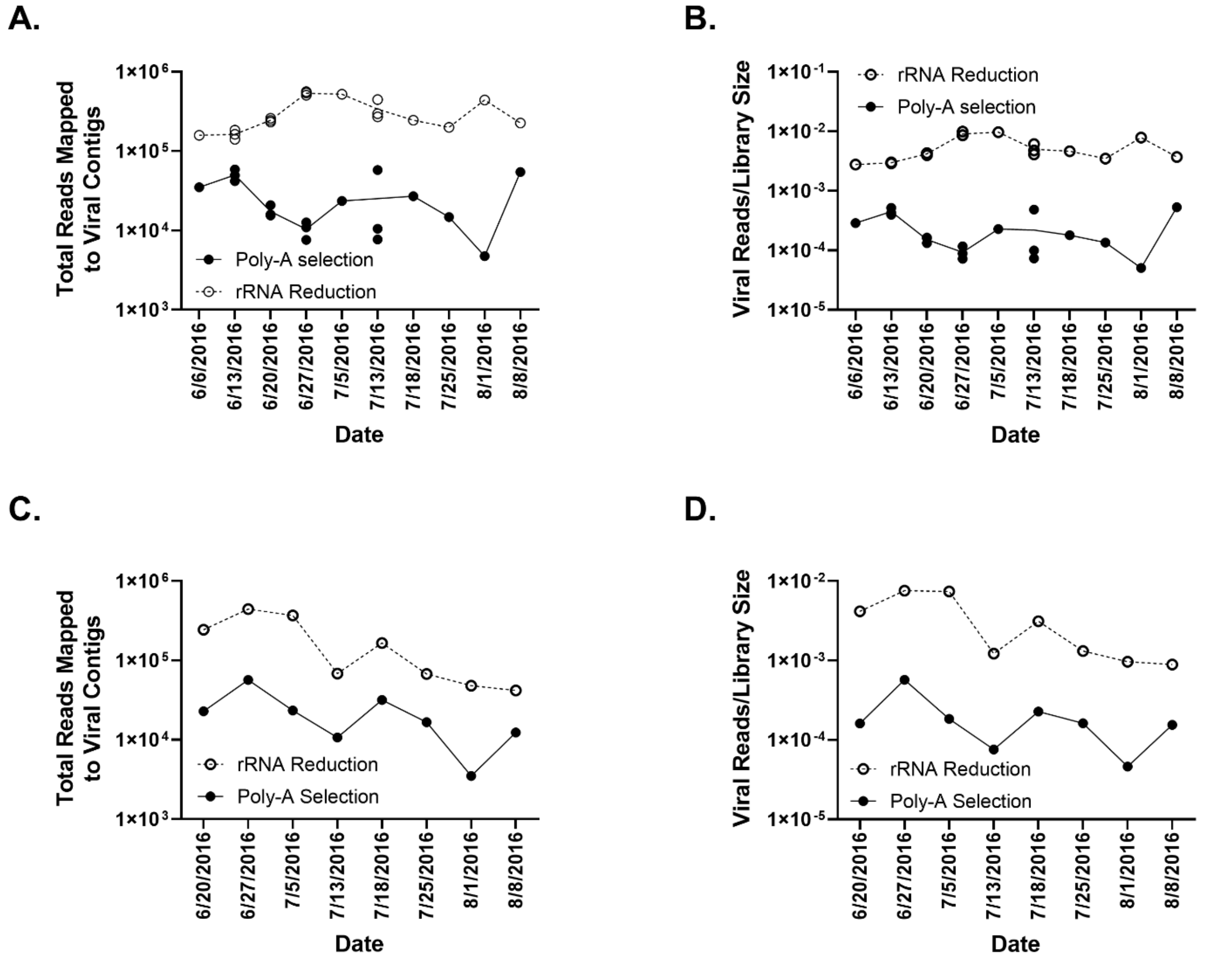

Supplemental Figure 4. Correlation between reads mapped to viral contigs in poly-A selected libraries v. rRNA-reduced libraries. Reads are normalized by dividing total reads mapped by the library size.

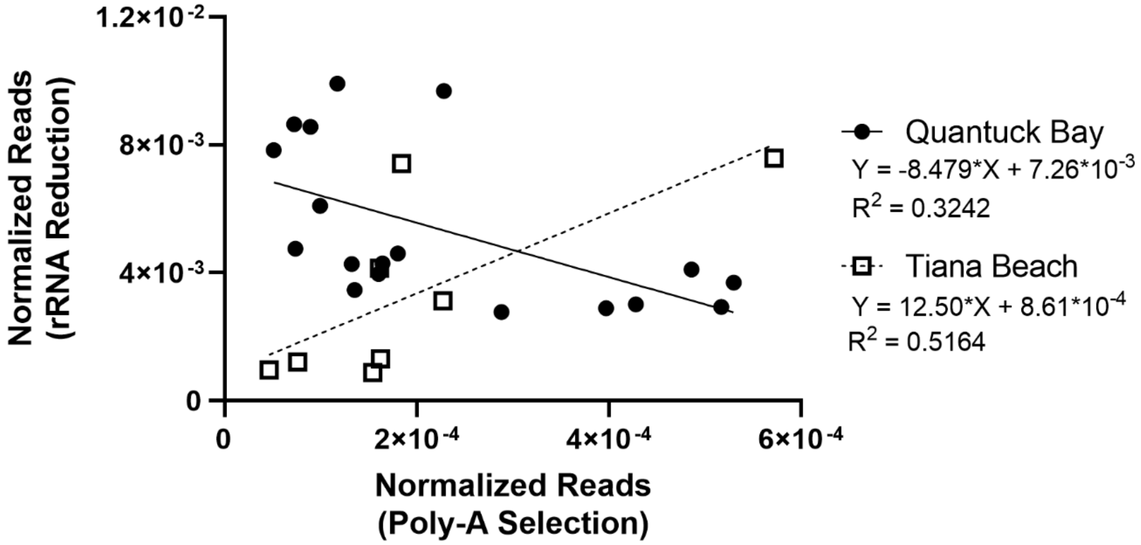

Supplemental Figure 5. Correlation between reads mapped to viral contigs with top BLASTx hits to the AaV genome v. reads mapped to the AaV genome. Reads are normalized by dividing total reads mapped by the library size.

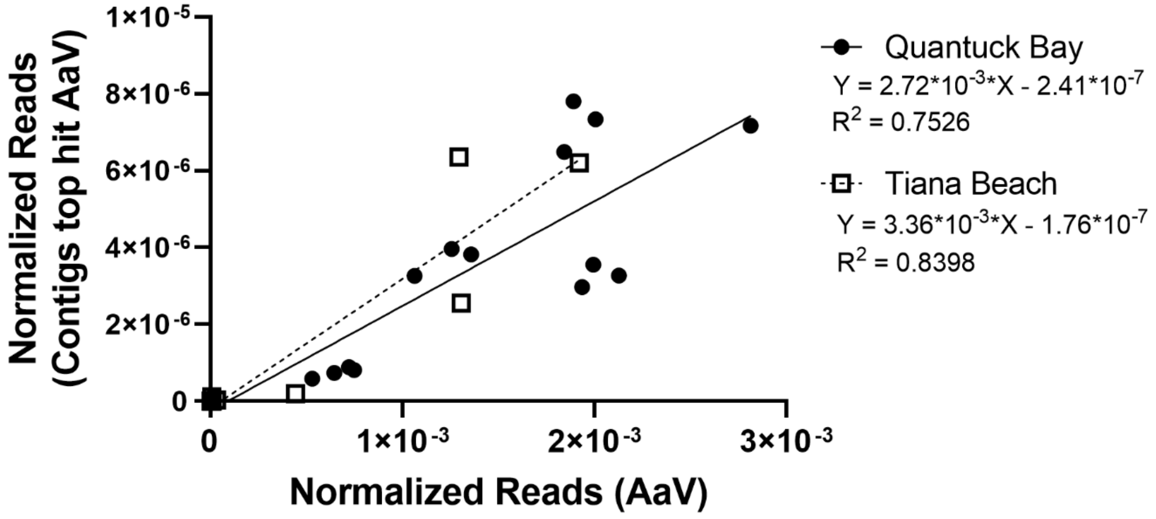

Supplemental Figure 6. Library Normalized Read Mappings to some viral families. Read Mappings to A) viruses infecting eukaryotes and B) viruses infecting prokaryotes. Reads are normalized by dividing total reads mapped by the library size and the length of the aligned portion of the contig. On dates where multiple libraries exist, all individual values are plotted.

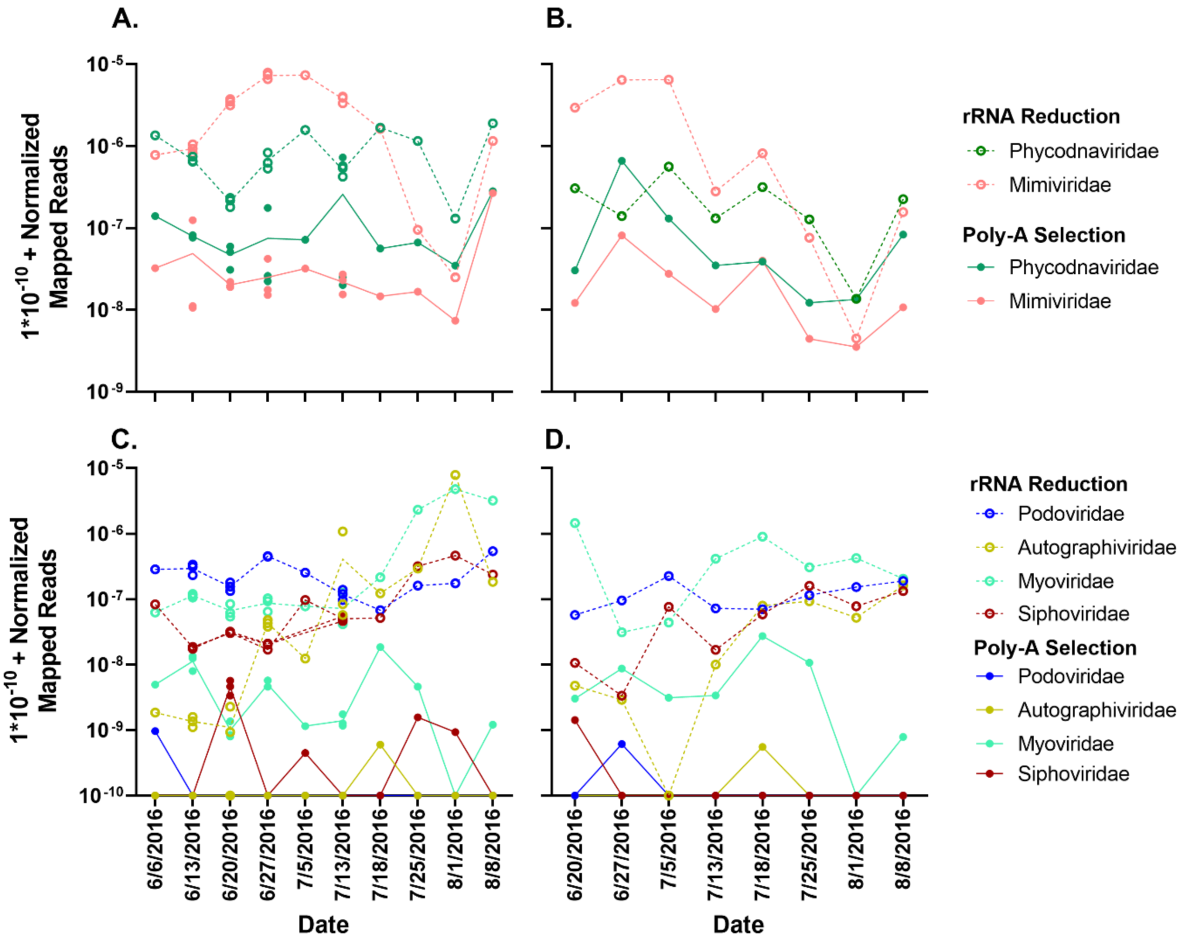

Supplemental Table 1. Description of Transcriptomic libraries generated in this study. [A] For normalized reads total reads mapped are normalized by library size.

Name	Date	Locations	Processing	Trimmed Reads	AaV Mapped Reads	AaV Normalized[A] Reads	*A. anophagefferens* Mapped reads	*A. anophagefferens* Normalized[A] reads
CM022	6/6/2016	Quantuck	Poly-A	1.22×10^{8}	1.68×10^{3}	1.38×10^{-5}	1.63×10^{6}	1.34×10^{-2}
CM107	6/6/2016	Quantuck	rRNA Reduced	5.70×10^{7}	3.01×10^{4}	5.29×10^{-4}	2.31×10^{5}	4.06×10^{-3}
CM023	6/13/2016	Quantuck	Poly-A	1.16×10^{8}	7.95×10^{2}	6.87×10^{-6}	1.19×10^{7}	1.03×10^{-1}
CM108	6/13/2016	Quantuck	rRNA Reduced	6.08×10^{7}	4.55×10^{4}	7.48×10^{-4}	1.77×10^{6}	2.91×10^{-2}
CM024	6/13/2016	Quantuck	Poly-A	1.14×10^{8}	8.15×10^{2}	7.16×10^{-6}	1.20×10^{7}	1.06×10^{-1}
CM109	6/13/2016	Quantuck	rRNA Reduced	5.53×10^{7}	3.56×10^{4}	6.44×10^{-4}	2.59×10^{6}	4.69×10^{-2}
CM025	6/13/2016	Quantuck	Poly-A	1.05×10^{8}	6.25×10^{2}	5.93×10^{-6}	1.10×10^{7}	1.04×10^{-1}
CM110	6/13/2016	Quantuck	rRNA Reduced	4.88×10^{7}	3.51×10^{4}	7.20×10^{-4}	1.25×10^{6}	2.57×10^{-2}
CM038	6/20/2016	Quantuck	Poly-A	1.17×10^{8}	1.48×10^{3}	1.27×10^{-5}	3.40×10^{7}	2.91×10^{-1}
CM123	6/20/2016	Quantuck	rRNA Reduced	5.68×10^{7}	1.21×10^{5}	2.13×10^{-3}	5.78×10^{6}	1.02×10^{-1}
CM039	6/20/2016	Quantuck	Poly-A	9.70×10^{7}	1.64×10^{3}	1.69×10^{-5}	2.83×10^{7}	2.92×10^{-1}
CM124	6/20/2016	Quantuck	rRNA Reduced	6.03×10^{7}	1.20×10^{5}	1.99×10^{-3}	7.04×10^{6}	1.17×10^{-1}
CM040	6/20/2016	Quantuck	Poly-A	1.30×10^{8}	1.80×10^{3}	1.38×10^{-5}	3.92×10^{7}	3.02×10^{-1}
CM125	6/20/2016	Quantuck	rRNA Reduced	5.86×10^{7}	1.13×10^{5}	1.93×10^{-3}	6.21×10^{6}	1.06×10^{-1}
CM056	6/27/2016	Quantuck	Poly-A	1.23×10^{8}	1.97×10^{3}	1.60×10^{-5}	3.65×10^{7}	2.98×10^{-1}
CM141	6/27/2016	Quantuck	rRNA Reduced	6.54×10^{7}	1.20×10^{5}	1.84×10^{-3}	7.46×10^{6}	1.14×10^{-1}
CM057	6/27/2016	Quantuck	Poly-A	1.08×10^{8}	1.13×10^{3}	1.05×10^{-5}	3.31×10^{7}	3.06×10^{-1}

CM142	6/27/2016	Quantuck	rRNA Reduced	5.43×10^{7}	1.03×10^{5}	1.89×10^{-3}	5.83×10^{6}	1.07×10^{-1}
CM058	6/27/2016	Quantuck	Poly-A	1.06×10^{8}	1.06×10^{3}	1.01×10^{-5}	3.27×10^{7}	3.09×10^{-1}
CM143	6/27/2016	Quantuck	rRNA Reduced	5.84×10^{7}	1.17×10^{5}	2.01×10^{-3}	6.39×10^{6}	1.09×10^{-1}
CM076	7/5/2016	Quantuck	Poly-A	1.03×10^{8}	1.30×10^{3}	1.26×10^{-5}	2.87×10^{7}	2.78×10^{-1}
CM161	7/5/2016	Quantuck	rRNA Reduced	5.37×10^{7}	1.51×10^{5}	2.82×10^{-3}	4.73×10^{6}	8.81×10^{-2}
CM077	7/13/2016	Quantuck	Poly-A	1.06×10^{8}	7.85×10^{2}	7.43×10^{-6}	2.17×10^{7}	2.05×10^{-1}
CM162	7/13/2016	Quantuck	rRNA Reduced	7.32×10^{7}	9.94×10^{4}	1.36×10^{-3}	4.69×10^{6}	6.41×10^{-2}
CM078	7/13/2016	Quantuck	Poly-A	1.05×10^{8}	7.06×10^{2}	6.75×10^{-6}	1.96×10^{7}	1.88×10^{-1}
CM163	7/13/2016	Quantuck	rRNA Reduced	5.70×10^{7}	7.15×10^{4}	1.26×10^{-3}	3.49×10^{6}	6.13×10^{-2}
CM079	7/13/2016	Quantuck	Poly-A	1.18×10^{8}	1.04×10^{3}	8.78×10^{-6}	2.09×10^{7}	1.76×10^{-1}
CM164	7/13/2016	Quantuck	rRNA Reduced	7.19×10^{7}	7.65×10^{4}	1.06×10^{-3}	3.57×10^{6}	4.96×10^{-2}
CM103	7/18/2016	Quantuck	Poly-A	1.50×10^{8}	6.49×10^{2}	4.33×10^{-6}	8.95×10^{5}	5.97×10^{-3}
CM188	7/18/2016	Quantuck	rRNA Reduced	5.31×10^{7}	7.25×10^{2}	1.36×10^{-5}	9.62×10^{4}	1.81×10^{-3}
CM104	7/25/2016	Quantuck	Poly-A	1.09×10^{8}	3.45×10^{2}	3.15×10^{-6}	7.50×10^{5}	6.86×10^{-3}
CM189	7/25/2016	Quantuck	rRNA Reduced	5.74×10^{7}	1.98×10^{2}	3.45×10^{-6}	1.16×10^{5}	2.03×10^{-3}
CM105	8/1/2016	Quantuck	Poly-A	9.35×10^{7}	5.31×10^{2}	5.68×10^{-6}	1.13×10^{6}	1.20×10^{-2}
CM190	8/1/2016	Quantuck	rRNA Reduced	5.62×10^{7}	6.90×10^{1}	1.23×10^{-6}	1.24×10^{5}	2.21×10^{-3}
CM106	8/8/2016	Quantuck	Poly-A	1.03×10^{8}	5.69×10^{2}	5.54×10^{-6}	4.13×10^{6}	4.03×10^{-2}
CM191	8/8/2016	Quantuck	rRNA Reduced	6.10×10^{7}	1.65×10^{2}	2.70×10^{-6}	5.00×10^{5}	8.19×10^{-3}
CM095	6/20/2016	Tiana	Poly-A	1.41×10^{8}	1.43×10^{3}	1.01×10^{-5}	2.17×10^{7}	1.54×10^{-1}
CM180	6/20/2016	Tiana	rRNA Reduced	5.85×10^{7}	7.64×10^{4}	1.31×10^{-3}	2.69×10^{6}	4.61×10^{-2}

CM096	6/27/2016	Tiana	Poly-A	9.91×10^{7}	1.48×10^{3}	1.49×10^{-5}	1.33×10^{7}	1.34×10^{-1}
CM181	6/27/2016	Tiana	rRNA Reduced	5.85×10^{7}	1.12×10^{5}	1.92×10^{-3}	2.03×10^{6}	3.48×10^{-2}
CM097	7/5/2016	Tiana	Poly-A	1.27×10^{8}	9.71×10^{2}	7.64×10^{-6}	2.11×10^{7}	1.66×10^{-1}
CM182	7/5/2016	Tiana	rRNA Reduced	4.97×10^{7}	6.43×10^{4}	1.29×10^{-3}	1.92×10^{6}	3.86×10^{-2}
CM098	7/13/2016	Tiana	Poly-A	1.41×10^{8}	8.57×10^{2}	6.06×10^{-6}	2.07×10^{7}	1.46×10^{-1}
CM183	7/13/2016	Tiana	rRNA Reduced	5.61×10^{7}	2.49×10^{4}	4.43×10^{-4}	2.08×10^{6}	3.70×10^{-2}
CM099	7/18/2016	Tiana	Poly-A	1.39×10^{8}	1.61×10^{3}	1.15×10^{-5}	4.21×10^{6}	3.02×10^{-2}
CM184	7/18/2016	Tiana	rRNA Reduced	5.31×10^{7}	1.64×10^{3}	3.09×10^{-5}	3.82×10^{5}	7.20×10^{-3}
CM100	7/25/2016	Tiana	Poly-A	1.03×10^{8}	4.77×10^{2}	4.63×10^{-6}	1.84×10^{6}	1.78×10^{-2}
CM185	7/25/2016	Tiana	rRNA Reduced	5.13×10^{7}	3.59×10^{2}	7.00×10^{-6}	1.74×10^{5}	3.39×10^{-3}
CM101	8/1/2016	Tiana	Poly-A	7.60×10^{7}	1.50×10^{2}	1.97×10^{-6}	2.10×10^{6}	2.77×10^{-2}
CM186	8/1/2016	Tiana	rRNA Reduced	4.97×10^{7}	1.63×10^{2}	3.28×10^{-6}	1.72×10^{5}	3.45×10^{-3}
CM102	8/8/2016	Tiana	Poly-A	8.02×10^{7}	3.95×10^{2}	4.92×10^{-6}	4.19×10^{6}	5.22×10^{-2}
CM187	8/8/2016	Tiana	rRNA Reduced	4.68×10^{7}	2.78×10^{2}	5.94×10^{-6}	3.74×10^{5}	7.99×10^{-3}

Supplemental Table 2. Description of poly-A selected transcriptomic libraries generated from a 2011 brown tide bloom in Quantuck Bay, NY (Moniruzzaman *et al* 2017, Wurch *et al.* 2019). [A] For normalized reads, total reads mapped are normalized by library size.

Transcriptome	Trimmed Reads	Number of Reads Mapped to AaV	[A]Normalized Reads to AaV
SRR3322145	40216623	684	1.70×10^{-5}
SRR3322387	41143577	1202	2.92×10^{-5}
SRR3322113	43741877	1761	4.03×10^{-5}
SRR3322397	39530226	1366	3.46×10^{-5}
SRR3322338	21928726	189	8.62×10^{-6}
SRR3322357	95527741	644	6.74×10^{-6}
SRR3322422	37433871	1178	3.15×10^{-5}
SRR3322410	36787907	1272	3.46×10^{-5}

Supplemental Table 3. Proportion of taxonomically classified reads from sampling date 6/27/2016.

Taxa	Poly-A average (SD)	rRNA average (SD)	Paired *t*-test p value	Taxa	Poly-A average (SD)	rRNA average (SD)	Paired *t*-test p value
Pelagophyceae	7.62×10^{-1} (1.30×10^{-2})	5.65×10^{-1} (5.55×10^{-3})	0.0044	Kinetoplastida	1.67×10^{-3} (1.46×10^{-5})	9.20×10^{-4} (2.01×10^{-5})	0.0011
Chlorophyta	4.33×10^{-2} (8.73×10^{-4})	4.99×10^{-2} (1.31×10^{-3})	0.0099	Perkinsea	1.64×10^{-3} (5.57E-05)	1.09×10^{-3} (4.46×10^{-5})	0.0066
Bacillariophyta	3.35×10^{-2} (9.79×10^{-4})	2.99×10^{-2} (1.34×10^{-3})	0.1516	Choanoflagellida	1.61×10^{-3} (4.52×10^{-5})	1.15×10^{-3} (1.98×10^{-5})	0.0098
Fungi	2.91×10^{-2} (2.31×10^{-3})	2.04×10^{-2} (4.26×10^{-4})	0.0335	Bangiophyceae	1.61×10^{-3} (2.36×10^{-5})	1.37×10^{-3} (1.68×10^{-5})	0.0008
Dinophyceae	2.25×10^{-2} (1.18×10^{-3})	1.83×10^{-2} (1.11×10^{-3})	0.0028	Mycetozoa	1.56×10^{-3} (2.00×10^{-5})	9.39×10^{-4} (1.66×10^{-5})	0.0016
Ciliophora	1.25×10^{-2} (1.16×10^{-3})	8.27×10^{-3} (3.69×10^{-4})	0.0378	Betaproteobacteria	1.54×10^{-3} (3.79×10^{-4})	5.03×10^{-3} (1.02×10^{-4})	0.0090
Oomycetes	8.78×10^{-3} (9.17×10^{-4})	5.34×10^{-3} (1.73×10^{-5})	0.0325	delta/epsilon subdivisions	1.48×10^{-3} (1.65×10^{-4})	3.79×10^{-3} (8.46×10^{-5})	0.0052
Isochrysidales	7.42×10^{-3} (1.05×10^{-4})	3.55×10^{-3} (6.99×10^{-5})	0.0002	Methanococci	1.43×10^{-3}) (3.48×10^{-5})	2.59×10^{-3} (1.15×10^{-4})	0.0035
Gammaproteobacteria	5.74×10^{-3} (1.52×10^{-3})	7.10×10^{-3} (1.29×10^{-3})	0.0006	uncultured bacterium	1.40×10^{-3} (1.04×10^{-3})	2.49×10^{-3} (1.14×10^{-5})	0.2797
Prymnesiales	5.60×10^{-3} (1.13×10^{-4})	3.57×10^{-3} (4.60×10^{-5})	0.0020	Euglenida	1.24×10^{-3} (3.21×10^{-5})	3.08×10^{-3} (7.34×10^{-5})	0.0003
PX clade	5.34×10^{-3} (3.65×10^{-4})	5.41×10^{-3} (6.17×10^{-5})	0.8322	Florideophyceae	1.23×10^{-3} (7.82×10^{-5})	3.22×10^{-3} (8.93×10^{-5})	0.0015
Pyrenomonadales	5.12×10^{-3} (1.88×10^{-4})	4.34×10^{-3} (1.76×10^{-5})	0.0333	Raphidophyceae	6.67×10^{-4} (8.17×10^{-6})	1.80×10^{-3} (8.04×10^{-5})	0.0026
Alphaproteobacteria	4.44×10^{-3} (1.07×10^{-3})	7.56×10^{-2} (1.45×10^{-3})	0.0005	Chrysophyceae	2.85×10^{-4} (1.43×10^{-5})	1.91×10^{-3} (6.98×10^{-5})	0.0008
Firmicutes	4.19×10^{-3} (1.56×10^{-3})	5.34×10^{-3} (5.85×10^{-4})	0.4554	Verrucomicrobia	2.39×10^{-4} (1.95×10^{-5})	2.72×10^{-3} (1.51×10^{-4})	0.0019

Cyanobacteria	4.00×10^{-3} (2.91×10^{-4})	9.53×10^{-3} (2.84×10^{-4})	0.0041	Phycodnaviridae	1.52×10^{-4} (1.38×10^{-5})	1.40×10^{-2} (3.83×10^{-4})	0.0004
Apicomplexa	3.99×10^{-3} (2.92×10^{-4})	2.19×10^{-3} (5.39×10^{-5})	0.0144	Synurophyceae	$9.57 \times 10^{-5})$ (5.96×10^{-6})	1.89×10^{-3} (4.95×10^{-5})	0.0003
Eustigmatophyceae	3.52×10^{-3} (1.64×10^{-5})	3.11×10^{-3} (4.19×10^{-5})	0.0059	Balneolia	9.34×10^{-5} (8.97×10^{-7})	2.66×10^{-3} (6.14×10^{-5})	0.0003
Actinobacteria	2.93×10^{-3} $(4.59 \times 10^{-4}$	4.98×10^{-3} (9.05×10^{-5})	0.0305	Dictyochophyceae	1.34×10^{-5} (1.30×10^{-6})	1.52×10^{-3} (2.88×10^{-5})	0.0002
Chromerida	2.54×10^{-3} $(3.90 \times 10^{-5}$	1.50×10^{-3} (6.84×10^{-5})	0.0004	Myoviridae	1.08×10^{-5} (1.28×10^{-6})	2.21×10^{-3} (1.05×10^{-5})	0.0011
Bacteroidetes	2.21×10^{-3} $(9.38 \times 10^{-5}$	3.72×10^{-2} (2.55×10^{-3})	0.0027				
Cercozoa	2.04×10^{-3} $(7.15 \times 10^{-5}$	1.28×10^{-3} (3.35×10^{-5})	0.0013				

Supplemental Table 4. Grouping of viral contigs by realm.

Realm	Poly-A Selection	rRNA Reduction
Varidnaviria	1481	12272
Duplodnaviria	85	6351
Riboviria	1094	5316
Monodnaviria	49	31
Unclassified	41	178
Unclassified DNA Viruses	19	68

Supplemental Table 5. Majority complete Riboviria contigs. Riboviria contigs were considered majority complete if the contig contained at least two unique aligned portions as determined by BLASTx, and there was a PFAM domain for a structural domain and an RDRP within the top BLASTx hits.

Library Type	Contig	Family	Length	Alignment Coordinates for Structural Domain (PFAM of top BLASTx hit)	Alignment Coordinates for RDRP Domain (PFAM of top BLASTx hit)
Poly-A selected	CM022_k119_232891	Unclassified	4667	1334:3214 (PF11492.8)	3614:4666 (PF00680.20)
Poly-A selected	CM022_k119_23982	Unclassified	10091	6880:8520 (PF08762.10)	2209:3051 (PF00680.20), 4123:6420 (PF00680.20)
Poly-A selected	CM022_k119_365009	Unclassified	4911	1958:3106 (PF11492.8)	62:1531 (PF00680.20)
Poly-A selected	CM022_k119_477777	Unclassified	5669	2968:4827 (PF00073.20)	775:2592 (PF00680.20)
Poly-A selected	CM023_k119_127985	Unclassified	10140	1808:2956 (PF11492.8)	3293:5089 (PF00680.20), 5942:7297 (PF00680.20)
Poly-A selected	CM023_k119_161727	Unclassified	8279	846:2705 (PF00073.20)	3081:4898 (PF00680.20)
Poly-A selected	CM023_k119_60873	Unclassified	10112	1571:3211 (PF08762.10)	3671:5968 (PF00680.20), 7040:7882 (PF00680.20)
Poly-A selected	CM024_k119_162980	Unclassified	6214	1808:2956 (PF11492.8)	3293:5089 (PF00680.20)
Poly-A selected	CM024_k119_23238	Unclassified	10112	6901:8541 (PF08762.10)	2230:3072 (PF00680.20), 4144:6441 (PF00680.20)
Poly-A selected	CM024_k119_293307	Unclassified	8276	846:2705 (PF00073.20)	3081:4898 (PF00680.20)
Poly-A selected	CM025_k119_132381	Unclassified	8269	834:2693 (PF00073.20)	3069:4886 (PF00680.20)
Poly-A selected	CM025_k119_25524	Unclassified	10115	6904:8544 (PF08762.10)	2233:3075 (PF00680.20), 4147:6444 (PF00680.20)
Poly-A selected	CM025_k119_263314	Unclassified	5643	2164:3312 (PF11492.8)	3649:5445 (PF00680.20)
Poly-A selected	CM038_k119_120416	Unclassified	9829	6874:8022 (PF11492.8)	2533:3888 (PF00680.20), 4741:6537 (PF00680.20)
Poly-A selected	CM038_k119_14618	Unclassified	8179	1568:3208 (PF08762.10)	3668:5965 (PF00680.20), 7037:7879 (PF00680.20)
Poly-A selected	CM038_k119_42632	Unclassified	8195	5547:7406 (PF00073.20)	3354:5171 (PF00680.20)
Poly-A selected	CM039_k119_110483	Unclassified	8259	5573:7432 (PF00073.20)	3380:5197 (PF00680.20)
Poly-A selected	CM039_k119_252260	Unclassified	3376	24:1172 (PF11492.8)	1509:3305 (PF00680.20)

Poly-A selected	CM039_k119_50818	Unclassified	8113	4908:6548 (PF08762.10)	2151:4448 (PF00680.20), 237:1079 (PF00680.20)
Poly-A selected	CM040_k119_39964	Unclassified	5714	3066:4925 (PF00073.20)	873:2690 (PF00680.20)
Poly-A selected	CM040_k119_84100	Unclassified	7827	4622:6262 (PF08762.10)	146:793 (PF00680.20), 1865:4162 (PF00680.20)
Poly-A selected	CM056_k119_130331	Unclassified	6474	1243:2892 (PF11492.8)	3801:6047 (PF00680.20)
Poly-A selected	CM056_k119_166663	Unclassified	5010	2377:4236 (PF00073.20)	184:2001 (PF00680.20)
Poly-A selected	CM056_k119_225589	Unclassified	4633	1680:2828 (PF11492.8)	48:1343 (PF00680.20)
Poly-A selected	CM057_k119_196821	Unclassified	4798	1240:2889 (PF11492.8)	3600:4778 (PF00680.20)
Poly-A selected	CM057_k119_197453	Unclassified	6883	3930:5078 (PF11492.8)	117:1121 (PF00680.20), 2034:3503 (PF00680.20)
Poly-A selected	CM057_k119_251203	Unclassified	5150	2474:4333 (PF00073.20)	281:2098 (PF00680.20)
Poly-A selected	CM058_k119_58160	Unclassified	5386	1187:2836 (PF11492.8)	3604:5376 (PF00680.20)
Poly-A selected	CM058_k119_96196	Unclassified	3966	1544:3184 (PF08762.10)	3605:3955 (PF00680.20)
Poly-A selected	CM076_k119_138192	Unclassified	9446	133:2037 (PF11492.8)	2565:4124 (PF00680.20)
Poly-A selected	CM076_k119_193048	Dicistroviridae	3749	873:3380 (PF11492.8)	2:562 (PF00680.20)
Poly-A selected	CM077_k119_202222	Unclassified	6942	4055:5704 (PF11492.8)	900:3146 (PF00680.20)
Poly-A selected	CM077_k119_26263	Picornavirales	8398	5924:7903 (PF11492.8)	1759:2571 (PF00680.20), 3817:5523 (PF00680.20)
Poly-A selected	CM079_k119_270436	Picornavirales	7038	4467:6446 (PF11492.8)	2360:4066 (PF00680.20), 302:1114 (PF00680.20)
Poly-A selected	CM079_k119_29068	Marnaviridae	5833	85:2694 (PF11492.8)	3069:4907 (PF00680.20)
Poly-A selected	CM079_k119_296173	Unclassified	5818	1229:2878 (PF11492.8)	3694:5817 (PF00680.20)
Poly-A selected	CM095_k119_44113	Unclassified	6020	1345:3225 (PF11492.8)	3625:5946 (PF00680.20)
Poly-A selected	CM096_k119_106060	Unclassified	4544	1344:3224 (PF11492.8)	3626:4543 (PF00680.20)
Poly-A selected	CM096_k119_242395	Unclassified	5956	1565:3205 (PF08762.10)	3590:5848 (PF00680.20)
Poly-A selected	CM096_k119_335584	Unclassified	8254	5577:7436 (PF00073.20)	3384:5201 (PF00680.20)
Poly-A selected	CM097_k119_175176	Unclassified	9554	245:2149 (PF11492.8)	2677:4236 (PF00680.20)
Poly-A selected	CM098_k119_338993	Unclassified	8022	285:2810 (PF11492.8)	3230:7273 (PF00680.20)
Poly-A selected	CM098_k119_423083	Unclassified	4543	1840:3699 (PF00073.20)	49:1509 (PF00680.20)
Poly-A selected	CM098_k119_450281	Unclassified	6710	3578:5173 (PF11492.8)	296:3298 (PF00680.20)
Poly-A selected	CM099_k119_339673	Marnaviridae	3545	270:2795 (PF11492.8)	3215:3544 (PF00680.20)

Poly-A selected	CM102_k119_124731	Marnaviridae	2241	1415:2239 (PF11492.8)	243:1403 (PF00680.20)
Poly-A selected	CM105_k119_130058	Unclassified	4446	365:2869 (PF11492.8)	3279:4445 (PF00680.20)
Poly-A selected	CM106_k119_111753	Unclassified	5511	2765:4435 (PF11492.8)	110:2305 (PF00680.20)
rRNA-reduced	CM107_k119_170491	Unclassified	10072	6905:8545 (PF08762.10)	2234:3076 (PF00680.20), 4148:6445 (PF00680.20)
rRNA-reduced	CM107_k119_18127	Unclassified	3797	859:3381 (PF11492.8)	3:563 (PF00680.20)
rRNA-reduced	CM107_k119_22876	Unclassified	8032	1732:2880 (PF11492.8)	3217:5013 (PF00680.20), 5866:7221 (PF00680.20)
rRNA-reduced	CM107_k119_265671	Marnaviridae	9584	252:2813 (PF11492.8)	2996:6082 (PF00680.20), 6488:8020 (PF00680.20)
rRNA-reduced	CM107_k119_280966	Unclassified	8175	201:2648 (PF11492.8)	3324:5198 (PF00680.20), 6564:8078 (PF00680.20)
rRNA-reduced	CM108_k119_130188	Marnaviridae	10708	1999:2802 (PF11492.8)	2833:4596 (PF00680.20)
rRNA-reduced	CM108_k119_13478	Marnaviridae	9579	6771:9332 (PF11492.8)	1564:3096 (PF00680.20), 3502:6588 (PF00680.20)
rRNA-reduced	CM108_k119_135553	Unclassified	8172	5525:7972 (PF11492.8)	2975:4849 (PF00680.20), 95:1609 (PF00680.20)
rRNA-reduced	CM108_k119_161465	Unclassified	1959	823:1848 (PF11492.8)	27:554 (PF00680.20)
rRNA-reduced	CM108_k119_174243	Unclassified	8118	24:1655 (PF11492.8)	1928:4033 (PF00680.20)
rRNA-reduced	CM108_k119_182773	Unclassified	10243	7056:8696 (PF08762.10)	2385:3227 (PF00680.20), 4299:6596 (PF00680.20)
rRNA-reduced	CM108_k119_188633	Unclassified	9865	489:3011 (PF11492.8)	3304:5949 (PF00680.20)
rRNA-reduced	CM108_k119_88173	Unclassified	7871	5022:6542 (PF11492.8)	142:1047 (PF00680.20), 2401:4488 (PF00680.20)
rRNA-reduced	CM109_k119_117460	Unclassified	3991	104:2206 (PF11492.8)	2777:3991 (PF00680.20)
rRNA-reduced	CM109_k119_132430	Marnaviridae	9583	251:2812 (PF11492.8)	2995:6081 (PF00680.20), 6487:8019 (PF00680.20)
rRNA-reduced	CM109_k119_170723	Unclassified	9862	1311:2831 (PF11492.8)	3365:5452 (PF00680.20), 6806:7711 (PF00680.20)
rRNA-reduced	CM109_k119_50054	Unclassified	8174	5527:7974 (PF11492.8)	2977:4851 (PF00680.20), 97:1611 (PF00680.20)
rRNA-reduced	CM109_k119_57876	Unclassified	9838	1741:3372 (PF11492.8)	3645:5750 (PF00680.20)

rRNA-reduced	CM109_k119_73587	Unclassified	10079	1537:3177 (PF08762.10)	3637:5934 (PF00680.20), 7006:7848 (PF00680.20)
rRNA-reduced	CM109_k119_89027	Unclassified	9882	6865:9387 (PF11492.8)	3927:6572 (PF00680.20)
rRNA-reduced	CM110_k119_110828	Unclassified	5348	1328:2848 (PF11492.8)	3322:5220 (PF00680.20)
rRNA-reduced	CM110_k119_39416	Unclassified	8052	1532:3172 (PF08762.10)	3632:5929 (PF00680.20), 7001:7843 (PF00680.20)
rRNA-reduced	CM110_k119_43542	Unclassified	9144	1042:2673 (PF11492.8)	2946:5051 (PF00680.20)
rRNA-reduced	CM110_k119_62991	Unclassified	4404	488:3010 (PF11492.8)	3306:4376 (PF00680.20)
rRNA-reduced	CM110_k119_89932	Marnaviridae	9579	251:2812 (PF11492.8)	2995:6081 (PF00680.20), 6487:8019 (PF00680.20)
rRNA-reduced	CM123_k119_136004	Unclassified	3417	611:3172 (PF11492.8)	3:428 (PF00680.20)
rRNA-reduced	CM123_k119_137271	Unclassified	9502	269:2788 (PF11492.8)	3724:8178 (PF00680.20)
rRNA-reduced	CM123_k119_42293	Unclassified	8804	5638:7278 (PF08762.10)	2881:5178 (PF00680.20), 967:1809 (PF00680.20)
rRNA-reduced	CM123_k119_65632	Unclassified	2314	63:1046 (PF11492.8)	1523:2308 (PF00680.20)
rRNA-reduced	CM123_k119_76029	Unclassified	8174	5527:7974 (PF11492.8)	2977:4851 (PF00680.20), 97:1611 (PF00680.20)
rRNA-reduced	CM124_k119_122389	Unclassified	9329	85:2604 (PF11492.8)	3540:7994 (PF00680.20)
rRNA-reduced	CM124_k119_131663	Unclassified	2329	25:1398 (PF11492.8)	1620:2327 (PF00680.20)
rRNA-reduced	CM124_k119_161817	Unclassified	10067	6899:8539 (PF08762.10)	2228:3070 (PF00680.20), 4142:6439 (PF00680.20)
rRNA-reduced	CM124_k119_46988	Unclassified	2666	961:1572 (PF00729.18)	2101:2655 (PF00998.23)
rRNA-reduced	CM124_k119_73272	Marnaviridae	4054	3201:4052 (PF11492.8)	16:3024 (PF00680.20)
rRNA-reduced	CM125_k119_105848	Unclassified	8632	288:1436 (PF11492.8)	1773:3569 (PF00680.20), 4422:5777 (PF00680.20)
rRNA-reduced	CM125_k119_10785	Marnaviridae	9570	239:2800 (PF11492.8)	2983:6069 (PF00680.20), 6475:8007 (PF00680.20)
rRNA-reduced	CM125_k119_162313	Unclassified	8807	1524:3164 (PF08762.10)	3624:5921 (PF00680.20), 6993:7835 (PF00680.20)
rRNA-reduced	CM125_k119_96646	Unclassified	2603	953:1564 (PF00729.18)	2108:2602 (PF00998.23)
rRNA-reduced	CM141_k119_12993	Unclassified	7355	1521:3161 (PF08762.10)	3621:5918 (PF00680.20)
rRNA-reduced	CM141_k119_132038	Unclassified	1157	780:1157 (PF11492.8)	1:606 (PF00680.20)
rRNA-reduced	CM141_k119_143491	Unclassified	4649	646:1794 (PF11492.8)	2131:3927 (PF00680.20)

rRNA-reduced	CM141_k119_23596	Unclassified	5611	1184:2833 (PF11492.8)	3601:5610 (PF00680.20)
rRNA-reduced	CM142_k119_45234	Unclassified	7781	4948:6597 (PF11492.8)	134:841 (PF00680.20), 1793:4039 (PF00680.20)
rRNA-reduced	CM142_k119_59507	Marnaviridae	2265	8:1528 (PF11492.8)	1705:2265 (PF00680.20)
rRNA-reduced	CM142_k119_79584	Unclassified	7162	1524:3164 (PF08762.10)	3624:5921 (PF00680.20)
rRNA-reduced	CM143_k119_182467	Unclassified	10060	6899:8539 (PF08762.10)	2228:3070 (PF00680.20), 4142:6439 (PF00680.20)
rRNA-reduced	CM143_k119_98386	Unclassified	9708	1187:2836 (PF11492.8)	3745:5991 (PF00680.20), 6961:8076 (PF00680.20)
rRNA-reduced	CM161_k119_140566	Unclassified	4149	569:2200 (PF11492.8)	2422:4032 (PF00680.20)
rRNA-reduced	CM161_k119_148120	Marnaviridae	6028	199:2760 (PF11492.8)	2943:5951 (PF00680.20)
rRNA-reduced	CM161_k119_160246	Unclassified	5014	3580:4992 (PF11492.8)	690:3335 (PF00680.20)
rRNA-reduced	CM161_k119_189387	Unclassified	9292	62:2581 (PF11492.8)	3517:7971 (PF00680.20)
rRNA-reduced	CM161_k119_192488	Unclassified	1384	554:1384 (PF11492.8)	1:288 (PF00680.20)
rRNA-reduced	CM161_k119_194120	Unclassified	8956	306:2768 (PF11492.8)	3048:7811 (PF00680.20)
rRNA-reduced	CM161_k119_194466	Unclassified	10065	1524:3164 (PF08762.10)	3624:5921 (PF00680.20), 6993:7835 (PF00680.20)
rRNA-reduced	CM161_k119_3952	Unclassified	9551	7408:9312 (PF11492.8)	5321:6880 (PF00680.20)
rRNA-reduced	CM162_k119_124509	Tombusviridae	4270	2781:3311 (PF00729.18)	910:2448 (PF00998.23)
rRNA-reduced	CM162_k119_1302	Unclassified	10058	1516:3156 (PF08762.10)	3616:5913 (PF00680.20), 6985:7827 (PF00680.20)
rRNA-reduced	CM162_k119_172352	Unclassified	2400	1892:2224 (PF11492.8)	1:1629 (PF00680.20)
rRNA-reduced	CM162_k119_201299	Unclassified	9691	274:2895 (PF11492.8)	3205:7758 (PF00680.20)
rRNA-reduced	CM162_k119_229707	Unclassified	1976	34:417 (PF11492.8)	1707:1976 (PF00680.20), 692:1687 (PF00680.20)
rRNA-reduced	CM162_k119_231412	Unclassified	8178	5528:7975 (PF11492.8)	2978:4852 (PF00680.20), 98:1612 (PF00680.20)
rRNA-reduced	CM162_k119_57771	Unclassified	9705	6886:8535 (PF11492.8)	1646:2761 (PF00680.20), 3731:5977 (PF00680.20)
rRNA-reduced	CM162_k119_71827	Marnaviridae	9581	6772:9333 (PF11492.8)	1565:3097 (PF00680.20), 3503:6589 (PF00680.20)
rRNA-reduced	CM163_k119_121408	Unclassified	9686	269:2890 (PF11492.8)	3200:7753 (PF00680.20)
rRNA-reduced	CM163_k119_166362	Tombusviridae	4089	2785:3315 (PF00729.18)	914:2452 (PF00998.23)

rRNA-reduced	CM163_k119_191923	Unclassified	10062	1521:3161 (PF08762.10)	3621:5918 (PF00680.20), 6990:7832 (PF00680.20)
rRNA-reduced	CM164_k119_105442	Unclassified	1457	1008:1457 (PF11492.8)	8:541 (PF00680.20)
rRNA-reduced	CM164_k119_125254	Unclassified	9716	1184:2833 (PF11492.8)	3742:5988 (PF00680.20), 6958:8073 (PF00680.20)
rRNA-reduced	CM164_k119_174117	Unclassified	1918	662:1918 (PF11492.8)	3:491 (PF00680.20)
rRNA-reduced	CM164_k119_192428	Unclassified	9691	274:2895 (PF11492.8)	3205:7758 (PF00680.20)
rRNA-reduced	CM164_k119_198269	Marnaviridae	9525	6767:9328 (PF11492.8)	1560:3092 (PF00680.20), 3498:6584 (PF00680.20)
rRNA-reduced	CM164_k119_62459	Tombusviridae	4249	936:1466 (PF00729.18)	1799:3337 (PF00998.23)
rRNA-reduced	CM164_k119_84880	Unclassified	10066	1530:3170 (PF08762.10)	3630:5927 (PF00680.20), 6999:7841 (PF00680.20)
rRNA-reduced	CM180_k119_113894	Unclassified	10066	1524:3164 (PF08762.10)	3624:5921 (PF00680.20), 6993:7835 (PF00680.20)
rRNA-reduced	CM180_k119_147837	Unclassified	9488	6788:9346 (PF11492.8)	1192:2781 (PF00680.20), 3709:6507 (PF00680.20)
rRNA-reduced	CM180_k119_19222	Unclassified	9208	321:2960 (PF11492.8)	3257:8137 (PF00680.20)
rRNA-reduced	CM180_k119_206477	Marnaviridae	8773	5768:8452 (PF11492.8)	926:5317 (PF00680.20)
rRNA-reduced	CM180_k119_74097	Unclassified	7707	3:1085 (PF11492.8)	1887:4082 (PF00680.20), 4773:6617 (PF00680.20)
rRNA-reduced	CM181_k119_131753	Unclassified	3498	2164:3483 (PF11492.8)	7:1593 (PF00680.20)
rRNA-reduced	CM181_k119_188690	Unclassified	9204	6585:8900 (PF11492.8)	1658:2599 (PF00680.20)
rRNA-reduced	CM181_k119_28089	Unclassified	8441	5278:6918 (PF08762.10)	2521:4818 (PF00680.20), 607:1449 (PF00680.20)
rRNA-reduced	CM181_k119_7847	Unclassified	9643	256:2796 (PF11492.8)	3077:5875 (PF00680.20), 6803:8392 (PF00680.20)
rRNA-reduced	CM182_k119_48313	Unclassified	5791	253:2826 (PF11492.8)	3188:5632 (PF00680.20)
rRNA-reduced	CM182_k119_90790	Unclassified	9515	7405:9309 (PF11492.8)	5318:6877 (PF00680.20)
rRNA-reduced	CM183_k119_167270	Unclassified	9200	301:2616 (PF11492.8)	6602:7543 (PF00680.20)
rRNA-reduced	CM183_k119_176451	Tombusviridae	4294	981:1511 (PF00729.18)	1844:3382 (PF00998.23)
rRNA-reduced	CM183_k119_192472	Unclassified	5210	2068:3708 (PF08762.10)	1:1695 (PF00680.20)
rRNA-reduced	CM183_k119_22885	Unclassified	2155	6:476 (PF11492.8)	757:2151 (PF00680.20)
rRNA-reduced	CM184_k119_116467	Unclassified	5070	3:2345 (PF11492.8)	2432:4780 (PF00680.20)

rRNA-reduced	CM184_k119_9777	Unclassified	9678	6795:9416 (PF11492.8)	1932:6485 (PF00680.20)
rRNA-reduced	CM185_k119_115175	Marnaviridae	10106	7114:9861 (PF08762.10)	2418:3467 (PF00680.20), 4281:6617 (PF00680.20)
rRNA-reduced	CM185_k119_40912	Totiviridae	5030	2995:4491 (PF05518.11)	9:1850 (PF02123.16)
rRNA-reduced	CM185_k119_84878	Totiviridae	5686	3800:5425 (PF05518.11)	39:2243 (PF02123.16)
rRNA-reduced	CM186_k119_124748	Unclassified	7370	270:2795 (PF11492.8)	3215:7258 (PF00680.20)
rRNA-reduced	CM186_k119_15381	Unclassified	9691	1345:2721 (PF11492.8)	4237:6204 (PF00680.20)
rRNA-reduced	CM186_k119_25617	Unclassified	6894	306:2762 (PF11492.8)	3079:6891 (PF00680.20)
rRNA-reduced	CM186_k119_88218	Unclassified	1829	57:968 (PF11492.8)	1240:1827 (PF00680.20)
rRNA-reduced	CM187_k119_166733	Unclassified	2068	38:901 (PF11492.8)	1119:2066 (PF00680.20)
rRNA-reduced	CM187_k119_36585	Marnaviridae	10107	245:2992 (PF08762.10)	3489:5825 (PF00680.20), 6639:7688 (PF00680.20)
rRNA-reduced	CM187_k119_64438	Unclassified	9683	6974:8350 (PF11492.8)	3491:5458 (PF00680.20)
rRNA-reduced	CM188_k119_12594	Unclassified	9670	263:2884 (PF11492.8)	3194:7747 (PF00680.20)
rRNA-reduced	CM189_k119_135932	Unclassified	5315	354:2858 (PF11492.8)	3268:5310 (PF00680.20)
rRNA-reduced	CM189_k119_176107	Unclassified	9691	1344:2720 (PF11492.8)	4236:6203 (PF00680.20)
rRNA-reduced	CM189_k119_182019	Marnaviridae	10107	245:2860 (PF08762.10)	3489:5825 (PF00680.20), 6639:7688 (PF00680.20)
rRNA-reduced	CM189_k119_84836	Marnaviridae	1070	4:489 (PF11492.8)	796:1068 (PF00680.20)
rRNA-reduced	CM190_k119_166905	Marnaviridae	9566	6766:9327 (PF11492.8)	1559:3091 (PF00680.20), 3497:6583 (PF00680.20)
rRNA-reduced	CM190_k119_2761	Unclassified	9390	1044:2420 (PF11492.8)	3936:5903 (PF00680.20)
rRNA-reduced	CM191_k119_114815	Marnaviridae	9919	7202:9817 (PF08762.10)	2374:3423 (PF00680.20), 4237:6573 (PF00680.20)
rRNA-reduced	CM191_k119_68271	Unclassified	1848	907:1770 (PF08762.10)	1:534 (PF00680.20)

Supplemental Table 6. Grouping of viral contigs by family.

Family	Poly-A Selection	rRNA Reduction
Mimiviridae	479	7847
Phycodnaviridae	902	4147
Unclassifed	952	3839
Podoviridae	2	2844
Myoviridae	69	1628
Autographiviridae	2	1060
Siphoviridae	9	771
Narnaviridae	21	719
Marnaviridae	73	313
Mitoviridae	0	219
Pacmanviridae	33	141
Totiviridae	2	99
Reoviridae	0	68
Circoviridae	44	23
Nodaviridae	0	61
Partitiviridae	14	43
Tombusviridae	0	56

Supplemental Table 7. Families of contigs with reciprocal BLASTn hits comparing poly-A selected and rRNA-reduced libraries.

Family	# Shared	Family	# Shared
Phycodnaviridae	246	Circoviridae	3
Unclassified	144	Baculoviridae	3
Mimiviridae	122	Poxviridae	2
Pacmanviridae	11	Pithoviridae	2
Marnaviridae	10	Iridoviridae	2
Dicistroviridae	10	Bacilladnaviridae	2
Narnaviridae	8	Qinviridae	1
Myoviridae	7	Pandoraviridae	1
Partitiviridae	5	Nudiviridae	1
Siphoviridae	4	Kaumoebaviridae	1
Marseilleviridae	3	Herelleviridae	1
Hypoviridae	3	Adintoviridae	1
Cruciviridae	3		

Supplemental Table 8. Paired t-test p values of the proportion of contigs and reads mapped by family by sample comparing poly-A selected and rRNA-reduced libraries. Bolded numbers indicate p values < 0.05.

Family	Proportion of Contigs	Proportion of Reads Mapped
Mimiviridae	**0.0002**	**<0.0001**
Phycodnaviridae	**<0.0001**	**<0.0001**
Myoviridae	**0.0025**	**0.0118**
Unclassifed	**<0.0001**	**0.0001**
Autographiviridae	**0.0003**	0.0656
Podoviridae	**<0.0001**	**<0.0001**
Siphoviridae	**<0.0001**	**0.0349**
Narnaviridae	**<0.0001**	**<0.0001**
Mitoviridae	**<0.0001**	**<0.0001**
Pacmanviridae	0.054	0.0611
Marnaviridae	**0.004**	0.0568
Nodaviridae	**<0.0001**	**0.0011**
Tombusviridae	**<0.0001**	**<0.0001**
Circoviridae	**<0.0001**	0.0634
Reoviridae	**0.0011**	0.07
Totiviridae	**<0.0001**	0.0588
Partitiviridae	**0.0164**	**0.0082**

Supplemental Table 9. Percentage of the number of reads mapped to top families in the poly-A selected v. rRNA-reduced libraries by sample.

Quantuck Bay

	Mimiviridae	Phycodnaviridae	Myoviridae	Autographiviridae	Podoviridae	Siphoviridae
Average	5.39	20.97	4.24	0.02	0.02	2.47
SD	8.87	37.33	3.79	0.09	0.07	5.52

Tiana Beach

	Mimiviridae	Phycodnaviridae	Myoviridae	Autographiviridae	Podoviridae	Siphoviridae
Average	12.67	85.95	5.31	0.08	0.07	0.29
SD	24.90	148.05	8.78	0.20	0.18	0.76
